# Streamlining psychosocial risk assessment: An exploratory adaptation of the COPSOQ III for Flemish healthcare workers

**DOI:** 10.1371/journal.pone.0342380

**Published:** 2026-02-05

**Authors:** Tahmineh Borhani, Hendrik Van Simaeys, Philippe Kiss, Els Clays

**Affiliations:** 1 Department of Politics and Philosophy, University of Idaho, Moscow, Idaho, United States of America; 2 Department of Public Health and Primary Care, Ghent University, Ghent, East Flanders, Belgium; Jonkoping University, SWEDEN

## Abstract

**Background:**

Healthcare workers are particularly vulnerable to psychosocial stress due to high emotional and cognitive demands, staffing shortages, and complex care responsibilities. The Copenhagen Psychosocial Questionnaire (COPSOQ III) is a widely used tool for assessing psychosocial risks at work, but its extended Flemish version has not been systematically evaluated in healthcare settings, and evidence on its psychometric performance in this context remains limited.

**Objective:**

This exploratory study aimed to examine the factor structure and internal consistency of the extended Flemish version of the COPSOQ III among healthcare workers in Flanders, Belgium, and to provide initial psychometric evidence.

**Methods:**

A cross-sectional survey was conducted among 242 employees across three healthcare institutions. Construct validity was examined through exploratory factor analysis (EFA) on polychoric correlations (oblimin rotation) to derive a revised factor structure of the extended Flemish COPSOQ III. Internal consistency of the resulting dimensions was assessed using Cronbach’s alpha, ordinal alpha, and McDonald’s omega (total and hierarchical). Multiple imputation was used in sensitivity analyses to evaluate the robustness of the factor structure and reliability estimates to missing data. A preliminary confirmatory factor analysis (CFA) was conducted as a supplementary, internal check of the proposed structure, rather than as a full confirmatory validation, given the limited sample size.

**Results:**

EFAs supported retention of the original COPSOQ III domain framework while indicating meaningful within-domain refinements, including merged, split, and reallocated dimensions; the number of dimensions was reduced from 45 to 34. Most resulting dimensions showed acceptable internal consistency (ordinal α and ω_total generally ≥ 0.70), although a small number of brief scales showed lower reliability. Sensitivity analyses using multiple imputation yielded highly similar factor solutions and reliability estimates. Domain-specific CFAs provided preliminary support for the revised structures with acceptable fit on commonly used indices (e.g., CFI/TLI, RMSEA, SRMR), but these results should be interpreted cautiously as internal checks rather than definitive confirmation.

**Conclusion:**

This exploratory study suggests that the extended Flemish version of COPSOQ III, with an adapted and more parsimonious structure, shows generally acceptable reliability and promising, though preliminary, evidence of construct validity for assessing psychosocial risks in healthcare settings. The adapted structure enhances its practical applicability while preserving theoretical integrity. Further research with larger and more diverse samples is recommended to confirm these findings in broader occupational contexts, test the stability of the proposed structure, and explore the utility of shorter versions in broader occupational contexts.

## Introduction

Work-related stress remains a pervasive issue across Europe and healthcare workers, in particular, are disproportionately affected by psychosocial stress due to the heavy workloads, emotional demands, and complex patient care responsibilities which significantly increase the risk of both mental and physical health problems [1,2]. Healthcare workers face not only significant physical challenges but also a considerable emotional burden, as they frequently confront morally and ethically difficult situations [3]. These challenges are further intensified by issues such as insufficient staffing, inadequate breaks, and the conflicting demands of balancing patient care with administrative duties [4]. Such stressors align with major psychosocial risk factors, including lack of support, diminished control over one’s work, and adverse working conditions [5,6]. The high prevalence of stress among healthcare workers underscores the urgent need for effective tools to assess and manage psychosocial risks in these environments.

The Copenhagen Psychosocial Questionnaire (COPSOQ) was developed as a comprehensive tool to measure work-related psychosocial risks, with applications in both occupational risk assessment and research on work and health [7]. In workplace settings, practitioners must assess a wide range of psychosocial factors at the organizational and national levels [8]. Research settings similarly require extensive coverage of these risk factors, which are central to work and health research. COPSOQ integrates key elements from prominent theoretical models that offer a foundational understanding of the psychosocial stressors that impact health, such as the Demand-Control Model [9], which explains job strain through the interaction of high demands and low control, and the Effort-Reward Imbalance Model [10], which focuses on the imbalance between the efforts workers exert and the rewards they receive. COPSOQ I was groundbreaking in capturing many aspects of the psychosocial work environment, making it applicable across sectors and occupations [7,11]. However, it did not address key work-related aspects such as rewards, equity, and trust. This gap led to the development of COPSOQ II [12,13]. Both COPSOQ I and II were available in short, medium, and long versions to cater to practical and research needs. Over time, it became evident that shorter versions could also meet research requirements, and the medium version proved to have sufficient reliability for this purpose [12,14].

The transition from COPSOQ II to COPSOQ III was driven by changes in the work environment, advances in psychosocial theories, and increased international usage [15]. Globalization, digitalization, and evolving management styles have altered working conditions, leading to increased precarious work, flexible schedules, and digital interactions. These changes necessitated the broader scope of COPSOQ III, which incorporates the Job Demands-Resources model, as well as emerging theories like Stress-as-Offence-to-Self, to provide a more comprehensive approach to understanding job demands, resources, productivity, and social capital [15–17].

Despite the extensive usage of COPSOQ in Flanders (Belgium), there remains a critical gap in the validation of the instrument. By doing so, it aims to contribute to the growing body of literature on occupational health tools and inform both researchers and practitioners about the utility of COPSOQ III in assessing psychosocial risks within healthcare settings.

## Materials and methods

### Study design and population

This exploratory psychometric study used a cross-sectional survey design to examine the internal consistency and factor structure of the extended Flemish version of the COPSOQ III among healthcare workers in Flanders. The survey was administered across multiple healthcare settings in Flanders over a two-month period, 20 November 2016–31 January 2017. The study population included a convenience sample of employees working in three distinct healthcare institutions, i.e., a specialized orthopedic rehabilitation and treatment facility with 324 employees and two residential care centers (one with 90 and one with 65 employees).

Employees received the questionnaire in envelopes distributed by their supervisors, along with a preliminary information letter, a consent form, and demographic questions. Completed questionnaires were returned in designated boxes placed in each unit. Response rates were monitored through visits to supervisors one week after the study began, followed by telephone interviews two weeks later, and all completed questionnaires were collected four weeks after the study began.

The study was approved by the Medical Ethics Committee of the University Hospital Ghent on 04 November 2016 (reference B670201629667). Participants were informed about the study’s purpose and methodology through the accompanying letter. Written informed consent was obtained by including the statement, “I agree to participate in this research: yes/no,” at the beginning of the questionnaire. No minors were enrolled in this study.

### Flemish COPSOQ III questionnaire

**Fig 1:**
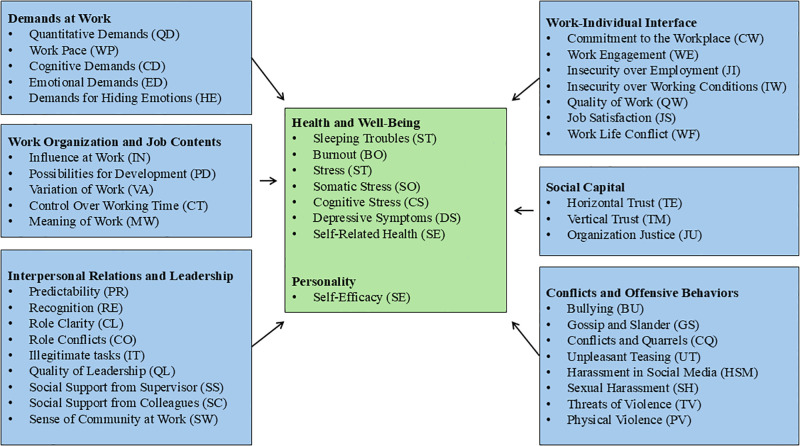
Overview of domains in the Flemish COPSOQ III long version.

The Flemish long version of COPSOQ III comprises eight domains—six for independent variables and two for dependent variables—encompassing 45 dimensions and 138 items in total (Fig 1). A mix of 5-point and 4-point Likert-type scales was used for item scoring. Each dimension score was calculated using standard methods, with all items contributing equally to the total, and the scores transformed to a 0–100 scale.

### Statistical analysis

Participants who left more than 15% of the COPSOQ questionnaire items unanswered within a given domain were excluded from analyses for that domain, following methodological guidelines. For scale scoring, if fewer than 15% of the items in a given dimension were missing, missing item scores were imputed using within-scale mean imputation, and the resulting dimension scores were transformed to a 0–100 scale, with higher scores reflecting a greater amount of the construct assessed in line with COPSOQ conventions. Descriptive statistics for the final scale scores and the amount of imputation applied are reported for each domain in the supplementary material (S1 Table).

We examined item-level patterns of missing data for all eight COPSOQ domains, and Little’s MCAR test was conducted to evaluate whether the missing data mechanism was compatible with a missing completely at random (MCAR) process. Across domains, item non-response was very low. Little’s MCAR tests showed a mixed pattern, with some domains yielding statistically significant results and others not (see S2 Table). Given the small proportion of missingness and the absence of strong, consistent evidence against MCAR across domains, we judged the missing data mechanism to be reasonably compatible with standard MCAR/MAR assumptions and therefore complemented the main analyses with multiple-imputation sensitivity analyses for ordinal items in each domain (described below).

First, construct validity was explored using exploratory factor analysis (EFA) within each COPSOQ III domain. EFA was based on polychoric correlation matrices estimated from all available pairs of observations (pairwise-complete) and was carried out using principal axis factoring with oblique (oblimin) rotation, reflecting the expected correlations between psychosocial constructs. The number of factors was guided by parallel analysis, scree plots, and interpretability. Data suitability for factor analysis was evaluated using the Kaiser–Meyer–Olkin (KMO) measure and Bartlett’s test of sphericity, with KMO values above 0.60 and a Bartlett test significance of p < 0.001 indicating adequate factorability [19]. Factor loadings of at least 0.40 were considered salient [18].

Second, Internal consistency of the EFA-derived dimensions was assessed using Cronbach’s alpha, ordinal alpha, and McDonald’s omega (total and hierarchical). As a general rule of thumb, values of ≥ 0.70 were considered acceptable for research purposes. We also examined mean scale scores, item-total correlations corrected for overlap, and the change in Cronbach’s alpha if an item was deleted to identify potentially problematic items.

Third, as a sensitivity analysis for the handling of missing data, we performed multiple imputation for ordinal item responses in each COPSOQ domain using multivariate imputation by chained equations (MICE) with predictive mean matching. For each domain, the imputation model included all items belonging to that domain together with three auxiliary variables (educational level, sex, and age). The number of imputations was determined separately for each domain based on the fraction of missing information and therefore varied slightly across domains; full details of the imputation settings are provided in the supplementary material (S3 Table). In each imputed dataset, polychoric correlations were re-estimated and the same EFA and reliability analyses described above were repeated, and parameter estimates were then pooled across imputations. Tucker’s factor congruence coefficients and comparisons of ordinal alpha and McDonald’s omega between the main and MI-pooled EFAs were used to evaluate the robustness of the factor structure and internal consistency estimates to the treatment of missing data.

Finally, as a limited, preliminary check of the EFA-derived structure, we conducted confirmatory factor analyses (CFAs) in a covariance-based structural equation modeling framework using a robust estimator appropriate for ordinal indicators. CFAs were estimated on listwise-complete cases, so that only respondents with complete data on the indicators in a given model were retained. Global model fit was evaluated using chi-square statistics, the comparative fit index (CFI), Tucker–Lewis index (TLI), root mean square error of approximation (RMSEA) with 90% confidence intervals, and the standardized root mean square residual (SRMR). In line with commonly used guidelines, values of CFI and TLI close to or above 0.90, RMSEA at or below 0.08, and SRMR at or below 0.08 were considered indicative of acceptable fit [20–22]. Given the modest sample size and the use of the same sample for both EFA and CFA, these CFAs were interpreted as preliminary internal checks rather than full confirmatory validations.

All analyses were conducted in R using RStudio. Exploratory factor analyses, reliability estimates, multiple imputation, and preliminary confirmatory factor analyses were implemented using established psychometric packages (e.g., psych, lavaan, semTools, and mice).

## Results

### Descriptive results and reliability

The study sample comprised 242 participants, including 49 men (20.2%) and 193 women (79.8%). Most participants (70.7%, n = 171) were employed in the orthopedic care facility, while 52 (21.5%) and 19 (7.9%) worked in residential care centers. Regarding age, 136 participants (56.2%) were under 45 years, 105 (43.4%) were aged 45 or older, and 1 (0.4%) did not disclose their age.

In terms of educational attainment, 133 participants (55.0%) had completed higher non-university education, 76 (31.4%) had completed higher secondary education, and 9 (3.7%) held a university degree. Additionally, 7 participants (2.9%) had post-secondary education, 14 (5.8%) had lower secondary education, and 1 (0.4%) had finished primary education. Two participants (0.8%) did not report their education level.

For occupational roles, 94 participants (38.8%) were nurses, 58 (24.0%) held managerial or directorial positions, 14 (5.8%) worked in administrative roles, and another 57 (23.6%) were employed in care or kitchen-related jobs. Eighteen participants (7.4%) reported other occupations. Regarding employment status, 120 participants (49.6%) were employed full-time, 66 (27.3%) worked between 76% and full-time, 52 (21.5%) between 50% and 75%, and 3 (1.2%) for less than 50% of full-time hours. One participant (0.4%) did not provide employment data.

### Construct validity: Exploratory factor analysis

Based on the KMO coefficient (>0.60) and Bartlett’s Test of Sphericity (p < 0.001), the data were deemed suitable for factor analysis. Detailed results from the exploratory factor analysis (EFA) within each of the eight COPSOQ III domains, based on polychoric correlation matrices and estimated using principal axis factoring with oblique (oblimin) rotation, are presented in (Table 1). Factor loadings of at least 0.40 were considered salient, and we prioritized items with primary loadings ≥ 0.50 and minimal cross-loadings when deciding which items to retain. The extracted factor solution revealed some deviations from the original structure, including the omission of items with cross-loadings, insufficient factor loadings, and single-item or regrouping of items.

**Table 1:** Exploratory factor analysis: Factor loadings (polychoric EFA, oblimin rotation).

Demands at work						
Items	Cognitive Demands (CD)	Quantitative Demands (QD)	Emotional & Decision Demands (EDD)	Demands for Hiding Emotions (HE)	Work Pace (WP)	
CD1	0.57					
CD2	0.82					
CD3	0.51					
QD1		0.84				
QD2		0.76				
QD4		0.52				
ED1			0.71			
ED2			0.54			
ED3			0.80			
CD4			0.50			
HE1				0.57		
HE3				0.56		
WP1					0.51	
WP2					0.84	
WP3					0.85	
Kaiser–Meyer–Olkin measure of sampling adequacy	Bartlett’s Test of Sphericity x2(105)	P < 0.001 (suitability for factor analysis)	Total Variance	N		
0.77	1406.76	P < 0.001	57.1	242		
Work Organization and Job Contents						
Items	Possibilities for Development (PD)	Workplace Autonomy (WA)	Influence at Work (IN)	Variation of Work (VA)	Meaning of Work (MW)	
PD1	0.86					
PD2	0.69					
PD3	0.83					
IN2		0.66				
IN3		0.66				
IN5		0.50				
CT4		0.67				
IN4			0.56			
IN6			0.94			
VA1				0.77		
VA2				0.80		
MW1					0.88	
MW2					0.95	
Kaiser–Meyer–Olkin measure of sampling adequacy	Bartlett’s Test of Sphericity x2(78)	P < 0.001 (suitability for factor analysis)	Total Variance	N		
0.77	1566.56	P < 0.001	65.55	242		
Interpersonal Relations and Leadership						
Items	Quality of Leadership (QL)	Sense of Community at Work (SW)	Role & Task Conflict (RT)	Role Clarity (CL)	Recognition (RE)	Social Support from Colleagues (SC)
QL1	0.75					
QL2	0.85					
Interpersonal Relations and Leadership						
QL3	0.65					
QL4	0.60					
SS3	0.50					
SW1		0.90				
SW2		0.87				
SW3		0.71				
CO1			0.79			
CO2			0.80			
IT1			0.61			
CL1				0.65		
CL2				0.86		
CL3				0.84		
RE1					0.66	
RE2					0.82	
RE3					0.75	
SC1						0.70
SC2						0.75
SC3						0.52
Kaiser–Meyer–Olkin measure of sampling adequacy	Bartlett’s Test of Sphericity x2(190)	P < 0.001 (suitability for factor analysis)	Total Variance	N		
0.89	3453.84	P < 0.001	69.70	242		
Work-Individual Interface						
Items	Commitment to the Workplace (CW)	Work Engagement (WE)	Insecurity Over Employment (JI)	Quality of Work (QW)	Work-Life Conflict (WF)	Insecurity Over Working Conditions (IW)
CW1	0.59					
CW2	0.79					
CW3	0.84					
CW4	0.60					
CW5	0.84					
WE1		0.94				
WE2		0.74				
WE3		0.85				
JI1			0.71			
JI2			0.61			
JI3			0.79			
QW1				0.89		
QW2				0.86		
WF1					0.52	
WF2					0.77	
WF3					0.81	
WF4					0.64	
WF5					0.80	
IW1						0.73
IW2						0.81
Work-Individual Interface						
IW3						0.81
IW4						0.70
Kaiser–Meyer–Olkin measure of sampling adequacy	Bartlett’s Test of Sphericity x2(231)	P < 0.001 (suitability for factor analysis)	Total Variance	N		
0.82	3768.09	P < 0.001	67.7	242		
Social Capital						
Items	Vertical Trust (TM)	Organizational Justice (JU)	Horizontal Trust (TE)			
TM1	0.79					
TM2	0.72					
TM4		0.64				
JU1		0.53				
JU2		0.84				
JU3		0.84				
JU4		0.52				
TE1			0.84			
TE2			0.95			
TM3			0.43			
Kaiser–Meyer–Olkin measure of sampling adequacy	Bartlett’s Test of Sphericity x2(45)	P < 0.001 (suitability for factor analysis)	Total Variance	N		
0.88	1243.60	P < 0.001	62.27	242		
Conflicts and Offensive Behaviors						
Items	Workplace Behavioral Transgression (WBT)	Violence and Harassment (VH)				
UT1	0.88					
BU1	0.94					
BU2	0.85					
SH1		0.68				
TV1		0.92				
PV1		0.87				
Kaiser–Meyer–Olkin measure of sampling adequacy	Bartlett’s Test of Sphericity x2(15)	P < 0.001 (suitability for factor analysis)	Total Variance			
0.65	1011.65	P < 0.001	74.00			
Health and Well-Being						
Items	Burnout (BO)	Stress (ST)	Sleeping Troubles (SL)	Somatic Stress (SO)	Cognitive Well-being Assessment (CWA)	
BO1	0.81					
BO2	0.82					
BO4	0.81					
ST2		0.54				
ST3		0.50				
SL1			0.75			
SL2			0.50			
SL3			0.94			
Health and Well-Being						
SL4			0.90			
SO1				0.69		
SO2				0.59		
SO3				0.55		
CS1					0.65	
CS2					0.92	
CS3					0.62	
CS4					0.88	
DS3					0.55	
Kaiser–Meyer–Olkin measure of sampling adequacy	Bartlett’s Test of Sphericity x2(136)	P < 0.001 (suitability for factor analysis)	Total Variance	N		
0.91	3097.22	P < 0.001	71.00	242		
Personality						
Items	Problem-Solving Self-Efficacy (PS)	Goal-Directed Self-Efficacy (GD)				
SE1	0.43					
SE4	0.66					
SE5	0.95					
SE6	0.67					
SE2		0.56				
SE3		0.70				
Kaiser–Meyer–Olkin measure of sampling adequacy	Bartlett’s Test of Sphericity x2(15)	P < 0.001 (suitability for factor analysis)	Total Variance	N		
0.84	547.85	P < 0.001	54.78	242		

In the **“Demands at Work”** domain, QD3 (from qualitative demands), HE2 and HE4 (from demands for hiding emotions) were excluded, and CD4 from the *cognitive demands* was regrouped with the emotional demands dimension and was renamed to “*Emotional and Decision Demands*” with a 4-item scale. KMO = 0.77 and Bartlett’s test (χ²(105) = 1406.76, p < 0.001) indicated adequate factorability. After removing and regrouping items as described above, EFA based on polychoric correlations supported a five-factor solution corresponding to Cognitive Demands (CD), Quantitative Demands (QD), Emotional & Decision Demands (EDD), Hiding Emotions (HE), and Work Pace (WP), with salient primary loadings ranging from 0.50 to 0.85 (Table 1). Internal consistency was acceptable to good across four of the five Demands subscales, with ordinal α ranging from 0.71 to 0.86 and ω_total from 0.74 to 0.87 (Table 2.1). In contrast, the two-item Demands for Hiding Emotions subscale showed lower reliability (ordinal α = 0.58 [0.41–0.70]; ω_total = 0.58 [0.42–0.70]), which is consistent with the very short length of this subscale; Hiding Emotions was therefore retained to preserve coverage of this content area, but its scores should be interpreted with some caution.

**Table 2:** 1. Ordinal alpha and McDonald’s ω for (total and hierarchical) Demands at Work (polychoric EFA). 2. Ordinal alpha and McDonald’s ω for (total and hierarchical) Work Organization and Job Contents (polychoric EFA). 3. Ordinal alpha and McDonald’s ω (total and hierarchical) for Interpersonal Relations and Leadership (polychoric EFA). 4. Ordinal alpha and McDonald’s ω for (total and hierarchical) Work–Individual Interface (polychoric EFA). 5. Ordinal alpha and McDonald’s ω (total and hierarchical) for Social Capital (polychoric EFA). 6. Ordinal alpha and McDonald’s ω (total and hierarchical) for Conflicts and Offensive Behaviors (polychoric EFA). 7. Ordinal alpha and McDonald’s ω (total and hierarchical) for Health and Well-being (polychoric EFA). 8. Ordinal alpha and McDonald’s ω for Personality (polychoric EFA).

1. Ordinal alpha and McDonald’s ω for (total and hierarchical) Demands at Work (polychoric EFA)				
Subscale (factor)	Items (n)	Ordinal α (95% CI)	ω_total (95% CI)	ω_hierarchical (95% CI)
Quantitative demands (QD)	3	0.85 [0.80–0.89]	0.85 [0.80–0.89]	0.85 [0.80–0.89]
Emotional & Decision Demands (ED)	4	0.79 [0.73–0.83]	0.80 [0.75–0.84]	0.80 [0.75–0.84]
Work pace (WP)	3	0.86 [0.82–0.90]	0.87 [0.84–0.90]	0.87 [0.84–0.90]
Cognitive demands (CD)	3	0.71 [0.62–0.77]	0.74 [0.66–0.79]	0.74 [0.66–0.79]
Demands for Hiding emotions (HE)	2	0.58 [0.41–0.70]	0.58 [0.42–0.70]	0.58 [0.42–0.70]
2. Ordinal alpha and McDonald’s ω for (total and hierarchical) Work Organization and Job Contents (polychoric EFA)				
**Subscale (factor)**	**Items (n)**	**Ordinal α (95% CI)**	**ω_total (95% CI)**	**ω_hierarchical (95% CI)**
Possibilities for development (PD)	3	0.90 [0.86–0.92]	0.90 [0.87–0.93]	0.90 [0.87–0.92]
Meaning of work (MW)	2	0.92 [0.89–0.96]	0.92 [0.88–0.96]	0.92 [0.88–0.96]
Workplace Autonomy (WA)	4	0.72 [0.65–0.78]	0.73 [0.65–0.79]	0.73 [0.65–0.79]
Variation of Work (VA)	2	0.79 [0.69–0.86]	0.79 [0.70–0.86]	0.79 [0.70–0.86]
Influence at Work (IN)	2	0.75 [0.66–0.83]	0.75 [0.65–0.83]	0.75 [0.65–0.83]
3. Ordinal alpha and McDonald’s ω (total and hierarchical) for Interpersonal Relations and Leadership (polychoric EFA)				
**Subscale (factor)**	**Items (n)**	**Ordinal α (95% CI)**	**ω_total (95% CI)**	**ω_hierarchical (95% CI)**
Quality of leadership (QL)	5	0.88 [0.84–0.90]	0.88 [0.85–0.90]	0.88 [0.85–0.90]
Recognition (RE)	3	0.92 [0.81–0.94]	0.92 [0.90–0.94]	0.92 [0.90–0.94]
Sense of community at work (SW)	3	0.93 [0.91–0.95]	0.93 [0.91–0.96]	0.93 [0.91–0.96]
Role & Task Conflict (RT)	3	0.89 [0.84–0.93]	0.89 [0.85–0.93]	0.89 [0.85–0.93]
Social support from colleagues (SC)	3	0.82 [0.77–0.86]	0.82 [0.76–0.86]	0.82 [0.76–0.86]
Role Clarity (CL)	3	0.76 [0.69–0.81]	0.80 [0.75–0.84]	0.80 [0.75–0.84]
4. Ordinal alpha and McDonald’s ω for (total and hierarchical) Work–Individual Interface (polychoric EFA)				
**Subscale (factor)**	**Items (n)**	**Ordinal α (95% CI)**	**ω_total (95% CI)**	**ω_hierarchical (95% CI)**
Commitment to the workplace (CW)	5	0.90 [0.87–0.92]	0.90 [0.88–0.92]	0.90 [0.88–0.92]
Work–life conflict (WF)	5	0.84 [0.80–0.87]	0.85 [0.81–0.88]	0.85 [0.81–0.88]
Insecurity over working conditions (IW)	4	0.88 [0.83–0.90]	0.88 [0.84–0.91]	0.88 [0.84–0.91]
Work engagement (WE)	3	0.94 [0.92–0.96]	0.94 [0.92–0.96]	0.94 [0.92–0.96]
Insecurity over employment (JI)	3	0.83 [0.77–0.86]	0.83 [0.78–0.88]	0.83 [0.78–0.88]
Quality of work (QW)	2	0.93 [0.89–0.96]	0.93 [0.90–0.96]	0.93 [0.90–0.96]
5. Ordinal alpha and McDonald’s ω (total and hierarchical) for Social Capital (polychoric EFA)				
**Subscale (factor)**	**Items (n)**	**Ordinal α (95% CI)**	**ω_total (95% CI)**	**ω_hierarchical (95% CI)**
Organizational Justice (JU)	5	0.90 [0.87–0.92]	0.90 [0.87–0.92]	0.90 [0.87–0.92]
Horizontal Trust (TE)	3	0.84 [0.79–0.89]	0.86 [0.82–0.90]	0.86 [0.82–0.90]
Vertical Trust (TM)	2	0.81 [0.73–0.88]	0.81 [0.73–0.87]	0.81 [0.73–0.87]
6. Ordinal alpha and McDonald’s ω (total and hierarchical) for Conflicts and Offensive Behaviors (polychoric EFA)				
**Subscale (factor)**	**Items (n)**	**Ordinal α (95% CI)**	**ω_total (95% CI)**	**ω_hierarchical (95% CI)**
Workplace Behavioral Transgression (WBT)	3	0.92 [0.86–0.95]	0.92 [0.86–0.95]	0.92 [0.86–0.95]
Violence and harassment (VH)	3	0.86 [0.74–0.92]	0.86 [0.79–0.93]	0.86 [0.79–0.93]
7. Ordinal alpha and McDonald’s ω (total and hierarchical) for Health and Well-being (polychoric EFA)				
**Subscale (factor)**	**Items (n)**	**Ordinal α (95% CI)**	**ω_total (95% CI)**	**ω_hierarchical (95% CI)**
Cognitive Well-being Assessment (CWA)	5	0.89 [0.87–0.92]	0.90 [0.87–0.92]	0.90 [0.87–0.92]
Burnout (BO)	3	0.95 [0.93–0.97]	0.95 [0.94–0.97]	0.95 [0.94–0.97]
Sleeping Troubles (SL)	4	0.89 [0.86–0.92]	0.90 [0.87–0.92]	0.90 [0.87–0.92]
Somatic Stress (SO)	3	0.79 [0.72–0.84]	0.79 [0.71–0.84]	0.79 [0.71–0.84]
Stress (ST)	2	0.92 [0.88–0.95]	0.92 [0.88–0.95]	0.92 [0.88–0.95]
8. Ordinal alpha and McDonald’s ω for Personality (polychoric EFA)				
**Subscale (factor)**	**Items (n)**	**Ordinal α (95% CI)**	**ω_total (95% CI)**	**ω_hierarchical (95% CI)**
Problem-Solving Self-Efficacy (PS)	4	0.84 [0.79–0.88]	0.85 [0.80–0.88]	0.85 [0.80–0.88]
Goal-Directed Self-Efficacy (GD)	2	0.63 [0.49–0.75]	0.63 [0.45–0.75]	0.63 [0.45–0.75]

In the **“Work Organization and Job Contents”** domain, IN1 (from influence at work), CT1, CT2, CT3, and CT5 (from control over working time), were excluded and CT4 from control over working time was regrouped with IN2, IN3, and IN5 from influence at work and was renamed to “*Workplace Autonomy*” with a 4-item scale referring to day-to-day control. The *influence at work* dimension was reduced to two items (IN4 and IN6) measuring influence over tasks and methods. KMO = 0.77 and Bartlett’s test (χ²(78) = 1566.56, p < 0.001) indicated that the data were suitable for factor analysis. EFA on polychoric correlations yielded a five-factor solution corresponding to Possibilities for Development (PD), Workplace Autonomy (WA), Influence at Work (IN), Variation of Work (VA), and Meaning of Work (MW), with primary loadings ranging from 0.50 to 0.95 (Table 1). Internal consistency was acceptable to excellent across all five Work Organization and Job Contents subscales, with ordinal α ranging from 0.72 (Workplace Autonomy) to 0.92 (Meaning of Work) and ω_total from 0.73 to 0.92 (Table 2.2). Workplace Autonomy was at the lower end of the acceptable range, whereas Meaning of Work, Possibilities for Development, and the two-item subscales for Variation of Work and Influence at Work all showed strong reliability despite their brevity.In the **“Interpersonal Relations and Leadership”** domain, SS1 and SS2 (from social support from supervisor), were excluded and SS3 social support from supervisor was regrouped with QL1-QL4 from quality of leadership to form a new 5-item “Quality of Leadership” scale capturing supportive and fair leadership behaviour. Additionally, two *role conflict* items (CO2, CO3) and one *illegitimate tasks* item were combined into “*Role and Task Conflict*.” KMO = 0.89 and Bartlett’s test of sphericity (χ²(190) = 3453.84, p < 0.001) indicated that the data were suitable for factor analysis. EFA on polychoric correlations yielded a six-factor solution corresponding to Quality of Leadership (QL), Recognition (RE), Sense of Community at Work (SW), Role & Task Conflict (RT), Social Support from Colleagues (SC), and a Role Clarity factor (CL), with primary loadings ranging from 0.50 to 0.90 and accounting for 69.7% of the total variance (Table 1). Internal consistency was acceptable to excellent across all six Interpersonal Relations and Leadership subscales, with ordinal α ranging from 0.76 (Role Clarity) to 0.93 (Sense of Community at Work) and ω_total from 0.80 to 0.93 (Table 2.3). Role Clarity was at the lower end of the acceptable range but still above conventional thresholds, whereas Sense of Community at Work and Recognition showed particularly high reliability.

In the “Work-Individual Interface” domain, IW5 (from insecurity over working conditions) and JS1-JS5 (from job satisfaction), were excluded. KMO = 0.87 and Bartlett’s test of sphericity (p < 0.001) indicated that the data were suitable for factor analysis. EFA on polychoric correlations yielded a six-factor solution corresponding to Commitment to the Workplace (CW), Work–Life Conflict (WF), Insecurity over Working Conditions (IW), Work Engagement (WE), Insecurity over Employment (JI), and Quality of Work (QW), with primary loadings ranging from 0.52 to 0.94 (Table 1). Internal consistency was acceptable to excellent across all six Work–Individual Interface subscales, with ordinal α ranging from 0.83 (Insecurity over Employment) to 0.94 (Work Engagement) and ω_total from 0.83 to 0.94 (Table 2.4). Insecurity over Employment was at the lower end of this range, whereas Work Engagement and the two-item Quality of Work subscale showed particularly high reliability, indicating that even the shortest scale provided a coherent indicator of its construct.In the **“Social Capital”** domain, TE3 (from horizontal trust), was excluded. Moreover, TM4 from vertical trust was regrouped with JU1-JU4 from organizational justice to form a new 5-item “*Organizational Justice*” scale that reflects employees’ opportunity for voice and fair treatment in organizational decision-making. Finally, TE1 and TE2 from *horizontal trust* were combined with TM3 from vertical trust to form a new 3-item “*Horizontal Trust”* scale, as they all refer to withholding or sharing of important information between employees and management. KMO = 0.88 and Bartlett’s test of sphericity (χ²(45) = 1243.60, p < 0.001) indicated that the data were suitable for factor analysis. EFA on polychoric correlations yielded a three-factor solution corresponding to Organizational Justice (JU), Horizontal Trust (TE), and Vertical Trust (TM), with primary loadings ranging from 0.43 to 0.95 and explaining 62.27% of the total variance (Table 1). Internal consistency was acceptable to excellent across all three Social Capital subscales, with ordinal α ranging from 0.81 (Vertical Trust) to 0.90 (Organizational Justice) and ω_total from 0.81 to 0.90 (Table 2.5). Vertical Trust, although based on only two items, still met conventional reliability thresholds, whereas Organizational Justice and the three-item Horizontal Trust scale showed particularly strong reliability.

In the **“Conflicts and Offensive Behaviors”** domain, GS1 (from gossip and slander), CQ1 (from Conflicts and quarrels), and HSM1 (from harassment in social media) were excluded. Moreover, the remaining dimensions and items were condensed into two: *Workplace Behavioral Transgression* (including: Bullying (BU1, BU2) and Unpleasant teasing (UT1)) and *Violence and Harassment* (including: Physical violence (PV1), Threats of violence (TV1), and Sexual harassment (SH1)). KMO = 0.65 and Bartlett’s test of sphericity (χ²(15) = 1011.65, p < 0.001) indicated that the Conflicts and Offensive Behaviors items were suitable for factor analysis, although the KMO value was somewhat lower than in other domains, which is expected given the small number of items. EFA on polychoric correlations yielded a clear two-factor solution corresponding to Workplace Behavioral Transgression (WBT) and Violence and Harassment (VH), with primary loadings ranging from 0.68 to 0.94 and accounting for 74.0% of the total variance (Table 1). Internal consistency was excellent for WBT (ordinal α = 0.92 [0.86–0.95]; ω_total = 0.92 [0.86–0.95]) and good for VH (ordinal α = 0.86 [0.74–0.92]; ω_total = 0.86 [0.79–0.93]) (Table 2.6), indicating that both subscales can be used as reliable indices of workplace transgressive behaviour and violence/harassment, respectively.

In the **“Health and Well-Being”** domain, GH1 (from self rated health), BO3 (from burnout), ST1 (from stress), SO4 (from somatic stress), DS1, DS2, and DS4 (from depressive symptoms) were excluded. Moreover, DS3 depressive symptoms was regrouped with CS1-CS4 from cognitive stress and was renamed to “Cognitive Well-being Assessment” with a 5-item scale referring to perceived cognitive strain and mentally burdensome thoughts in everyday functioning.

KMO = 0.91 and Bartlett’s test of sphericity (χ²(136) = 3097.22, p < 0.001) indicated that the Health and Well-Being items were suitable for factor analysis. EFA on polychoric correlations yielded a five-factor solution corresponding to CWA, Burnout (BO), Sleeping Troubles (SL), Somatic Stress (SO), and Stress (ST), with primary loadings ranging from 0.50 to 0.94 and accounting for 71.0% of the total variance (Table 1). Internal consistency was acceptable to excellent across all five Health and Well-being subscales, with ordinal α ranging from 0.79 (Somatic Stress) to 0.95 (Burnout) and ω_total from 0.79 to 0.95 (Table 2.7). Somatic Stress was at the lower end of this range but still met conventional reliability thresholds, whereas Burnout and the two-item Stress subscale showed particularly high reliability, indicating that even the brief Stress scale provides a coherent indicator of perceived stress.

Within the “Personality” domain, the original self-efficacy scale split into two related but distinct subdimensions based on parallel analysis and EFA. Items SE1, SE4, SE5, and SE6 loaded on a “*Problem-Solving Self-Efficacy*” factor, reflecting confidence in handling problems and finding solutions, whereas items SE2 and SE3 loaded on a “*Goal-Directed Self-Efficacy*” factor, capturing confidence in pursuing and achieving personal goals. KMO = 0.84 and Bartlett’s test of sphericity (χ²(15) = 547.85, p < 0.001) indicated that the items were suitable for factor analysis. EFA on polychoric correlations yielded a two-factor solution corresponding to Problem-Solving Self-Efficacy (PS) and Goal-Directed Self-Efficacy (GD), with primary loadings ranging from 0.43 to 0.95 and accounting for 54.78% of the total variance (Table 1). Internal consistency was good for PS (ordinal α = 0.84 [0.79–0.88]; ω_total = 0.85 [0.80–0.88]; ω_hierarchical = 0.85 [0.80–0.88]), indicating a largely unidimensional problem-solving self-efficacy factor (Table 2.8). Reliability for GD was more modest (ordinal α = 0.63 [0.49–0.75]; ω_total = 0.63 [0.45–0.75]; ω_hierarchical = 0.63 [0.45–0.75]), which is partly expected for a very short two-item subscale; GD was therefore retained to capture this theoretically distinct aspect of self-efficacy, but its scores should be interpreted with some caution in subsequent applications.

### Sensitivity analysis: Missing data and multiple imputation

Across domains, item non-response was very low (generally below a few percent per item), and Little’s MCAR tests did not provide strong evidence against a MCAR/MAR mechanism (S2 Table). To examine the robustness of our findings to the handling of missing data, we repeated the EFA and reliability analyses in each domain on multiply imputed datasets generated with chained equations (MICE) implemented in R for ordinal items, using domain-specific imputation settings. Imputations were run via the mifa wrapper around mice, and results were pooled using Rubin’s rules. For each domain, the imputation model included all items belonging to that domain together with three auxiliary variables (educational level, sex, and age). The number of imputations varied by domain to ensure stable pooled estimates given domain-specific missingness and model convergence. The MI-pooled diagnostics (e.g., KMO, Bartlett’s tests and parallel analyses) supported the same number of factors as in the main analyses (see S3 Table), and the factor loading patterns were highly similar, as reflected by high Tucker’s factor congruence coefficients between the main and MI-pooled EFAs (S4 Table). Ordinal alpha and McDonald’s ω were also very similar across approaches, with only minor differences in point estimates and overlapping confidence intervals (S5 Table). These results indicate that the extracted factor structures and reliability estimates are robust to the treatment of missing data, so we present the main (non-imputed) EFA and reliability results in the primary tables (Table 1).

### Preliminary confirmatory factor analysis

Preliminary CFA for the “Demands at Work” domain supported the five-factor solution (Quantitative Demands, Work Pace, Cognitive Demands, Emotional & Decision Demands, and Demands for Hiding Emotions). Global fit indices were generally acceptable (CFI/TLI ≈ 0.97/0.96, SRMR ≈ 0.08), with RMSEA slightly above the conventional 0.08 cut-off (≈0.09) but still within a range often interpreted as indicating reasonable fit when other indices are strong (see CFA tables for Demands at Work, S6 Table, 6_1_1–6_1_4). Reliability and AVE were clearly adequate for all factors, whereas Emotional & Decision Demands (EDD) and Hiding Emotions (HE) showed somewhat weaker convergent validity, with AVE just below 0.50 despite acceptable internal consistency (ordinal α and ω around the mid-0.70s for EDD and around 0.58 for the very short two-item HE scale). For HE, the combination of only two items and moderate standardized loadings (≈0.56–0.57) naturally limits the AVE, and for EDD the items capture several closely related facets of emotional and decisional strain, which can dilute AVE while still forming a coherent factor. Given the theoretically important content of these dimensions in COPSOQ and the evidence from Fornell–Larcker and HTMT indices that the five Demands factors remain clearly distinguishable from one another, we retained all five as separate factors, while recommending some caution in interpreting scores on Hiding Emotions.

Preliminary CFA for the “Work Organization and Job Contents” domain supported the five-factor solution (Possibilities for Development, Workplace Autonomy, Influence at Work, Variation of Work, and Meaning of Work), with global fit indices in the acceptable to excellent range (CFI/TLI ≥ 0.90, RMSEA and SRMR ≤ 0.08; see CFA tables for Work Organization and Job Contents, S6 Table, 6_2_1–6_2_4). While reliability and AVE were clearly adequate for all factors, Workplace Autonomy showed somewhat weaker convergent validity, with AVE below the conventional 0.50 threshold despite acceptable internal consistency (ordinal α and ω in the low- to mid-0.70s). This pattern is consistent with its moderate standardized loadings (around 0.50–0.67) and the fact that WA intentionally captures several facets of day-to-day autonomy (e.g., influence over colleagues, allocation of work, how work is carried out, and the possibility to leave work for private matters), which can dilute AVE while preserving a coherent construct. Given its theoretical importance in COPSOQ and the evidence from discriminant validity indices (Fornell–Larcker and HTMT) that WA remains clearly distinguishable from neighbouring factors, we retained Workplace Autonomy as a separate factor.

Preliminary CFA for the “Interpersonal Relations and Leadership” domain supported the six-factor solution (Quality of Leadership, Recognition, Sense of Community at Work, Role & Task Conflict, Social Support from Colleagues, and Role Clarity), with excellent global fit (CFI/TLI ≈ 1.00, RMSEA < 0.04, SRMR ≤ 0.06; see CFA tables for Interpersonal Relations and Leadership, S6 Table, 6_3_1–6_3_4). Internal consistency and convergent validity were good to excellent across all subscales (ordinal α and ω ≥ 0.79, AVE ≈ 0.64–0.92), indicating well-defined latent constructs. Fornell–Larcker and HTMT indices further supported discriminant validity, with the square roots of AVE exceeding the interfactor correlations and HTMT estimates remaining clearly below 0.85 with confidence intervals not approaching 1.00.

Preliminary CFA for the “Work–Individual Interface” domain supported the six-factor solution (Commitment to the Workplace, Work–Life Conflict, Insecurity over Working Conditions, Work Engagement, Insecurity over Employment, and Quality of Work), with global fit indices in the acceptable to excellent range (CFI/TLI ≥ 0.90, RMSEA and SRMR ≤ 0.08; see CFA tables for Work–Individual Interface, S6 Table, 6_4_1–6_4_4). Reliability and AVE were clearly adequate to excellent for all six subscales (ordinal α and ω generally ≥ 0.80 and AVE ≥ 0.62), indicating good convergent validity. Fornell–Larcker criteria and HTMT ratios also supported discriminant validity, as the square roots of AVE exceeded inter-factor correlations and HTMT estimates remained well below the conservative 0.85 threshold.

Preliminary CFA for the “Social Capital” domain supported the three-factor solution (Organizational Justice, Horizontal Trust, and Vertical Trust), with global fit indices in the acceptable to excellent range (CFI/TLI ≥ 0.90, RMSEA and SRMR ≤ 0.08; see CFA tables for Social Capital, S6 Table, 6_5_1–6_5_4). Reliability and convergent validity indices were very good for all three factors, with ordinal α and McDonald’s ω ≥ 0.80 and AVE values above 0.60, indicating substantial common variance among the items. Although Vertical Trust is based on only two items, its α/ω around 0.80 and AVE well above the 0.50 threshold suggest that this brief scale still forms a coherent indicator of perceived trust in management, albeit with a somewhat narrower content coverage. Fornell–Larcker and HTMT statistics further supported discriminant validity, as the square roots of AVE exceeded inter-factor correlations and HTMT confidence intervals remained clearly below 0.85.

Preliminary CFA for the “Conflicts and Offensive Behaviors” domain supported the expected two-factor solution—Workplace Behavioral Transgression (WBT) and Violence and Harassment (VH)—with excellent global fit (χ²/df < 1, CFI/TLI ≈ 1.00, RMSEA ≈ 0, SRMR ≈ 0.07; see CFA tables for Conflicts and Offensive Behaviors, S6 Table, 6_6_1–6_6_4). Reliability and convergent validity were clearly satisfactory for both subscales: ordinal α ranged from 0.86 to 0.92, McDonald’s ω from about 0.77 to 0.80, and AVE values were high (≈0.69–0.79), reflecting the strong standardized loadings of the three items per factor. Discriminant validity was also well supported, with the square roots of AVE exceeding the inter-factor correlation and the HTMT ratio (≈0.14, 95% CI clearly below 0.85) indicating that WBT and VH are empirically distinct.

Preliminary CFA for the “Health and Well-being” domain supported the five-factor solution (Cognitive Well-being Assessment, Burnout, Sleeping Troubles, Somatic Stress, and Stress), with excellent global fit (CFI/TLI ≈ 0.99, RMSEA < 0.05 with a narrow 90% CI, SRMR < 0.06; see CFA tables for Health and Well-being, S6 Table, 6_7_1–6_7_4). Internal consistency and convergent validity were generally very strong across all five factors (ordinal α and ω mostly in the high.80s–.90s, AVE ≥ 0.56). Discriminant validity was also supported by the Fornell–Larcker and HTMT criteria, although Somatic Stress (SO) and Stress (ST) showed a relatively high latent correlation (around r = 0.77) and borderline Fornell–Larcker results, indicating substantial but still acceptable overlap between these two symptom clusters (SO–ST HTMT values remained below the conservative 0.85 cutoff). Given the theoretical distinction between somatic stress symptoms and more general stress perceptions, and the consistently strong item loadings, both Somatic Stress and Stress were retained as closely related but distinct factors.

Preliminary CFA for the “Personality” domain supported the intended two-factor structure (Problem-Solving Self-Efficacy, Goal-Directed Self-Efficacy), with excellent global fit (χ²/df ≈ 1.13, CFI/TLI ≈ 0.99, RMSEA ≈ 0.02, SRMR ≈ 0.04; see CFA tables for Personality, S6 Table, 6_8_1–6_8_4). Problem-Solving Self-Efficacy (PS) showed good properties for a very short subscale, with acceptable internal consistency (ordinal α ≈ 0.85, ω ≈ 0.77) and AVE around 0.60, indicating adequate convergent validity. In contrast, Goal-Directed Self-Efficacy (GD) exhibited weaker convergent validity (α ≈ 0.63, ω ≈ 0.54, AVE ≈ 0.46), which is not unexpected for a two-item factor and suggests that scores on this subscale should be interpreted with some caution. Discriminant validity between the two factors was somewhat borderline: the square root of AVE for GD (≈ 0.68) was similar to its correlation with PS (≈ 0.74), and the HTMT estimate was 0.75 (95% CI 0.57–0.94). Overall, these results suggest that PS and GD are strongly related facets of a broader self-efficacy construct, but still empirically distinguishable; we therefore retained both as separate preliminary factors.

## Discussion

The COPSOQ instrument is widely recognized and extensively used in research for assessing psychosocial risks in workplaces [15]. Currently translated into 18 languages and utilized in 40 countries, its development is overseen by the International COPSOQ Network (www.copsoq-network.org). For COPSOQ I and II, the Flemish translations were conducted by Securex occupational physicians, involving a meticulous process of translation and back-translation into Danish, with validation by the original developers. In contrast, for COPSOQ III, additional Flemish translations were performed without back-translation. This study evaluated the psychometric properties of the extended Flemish COPSOQ III long version in a sample of workers in healthcare—a group known to experience unique psychosocial stressors, including high emotional and quantitative work demands, warranting specialized tools for risk assessment [23]. A convenience sample of 242 employees from three distinct healthcare institutions participated in this study, which facilitated the evaluation of COPSOQ III across various healthcare settings. By including employees from three different healthcare institutions and a range of professional roles, we were able to examine how well this version captures a broad range of psychosocial risk factors across diverse healthcare roles and settings.

Overall, the extended Flemish COPSOQ III showed generally acceptable psychometric performance in this sample. Across all eight domains, the data met commonly used criteria for factorability (KMO > 0.60, Bartlett’s test p < 0.001), and EFAs based on polychoric correlations largely supported the international COPSOQ III framework while suggesting several meaningful refinements. Items with weak or cross-loadings were removed, and some scales were reconfigured into more coherent subdimensions. Most subscales demonstrated acceptable to excellent internal consistency, with ordinal α and McDonald’s ω typically at or above 0.70, and convergent validity indices (AVE) at or above the conventional 0.50 benchmark for the majority of dimensions.

Beyond overall fit and reliability, our EFAs showed that the Flemish healthcare version of COPSOQ III largely preserved the original domain structure while adapting the composition of several dimensions. At the domain level, all eight COPSOQ III domains were retained, and many core dimensions kept their original meaning with only minor item deletions. Other scales were recomposed into more coherent subdimensions within the same domains: for example, emotional demand items with one item of cognitive demands were combined and were relocated into “Emotional & Decision Demands”, items from influence at work and one item from control over working time were reorganized into “Workplace Autonomy”, leadership items and one item from supervisor-support items were merged into a broader “Quality of Leadership” scale, several trust and fairness items were reallocated into “Organizational Justice”, “Horizontal Trust”, and “Vertical Trust”, and the original self-efficacy scale split into “Problem-Solving Self-Efficacy” and “Goal-Directed Self-Efficacy”. In addition, new composite scales such as “Workplace Behavioral Transgression”, “Violence and Harassment”, and “Cognitive Well-being Assessment” emerged to capture patterns that were particularly salient in this Flemish healthcare sample. A detailed, dimension-level mapping of all retained, shortened, merged, split, reallocated, and dropped dimensions is provided in S7 Table. This mapping summarizes the empirical basis for the revisions (e.g., salient primary loadings and problematic cross-loadings) alongside within-domain conceptual coherence, and should be interpreted as a theoretically informed, exploratory proposal for future testing rather than a finalized validation. Importantly, content coverage is largely preserved at the domain level: all eight COPSOQ III domains remain represented, and most core constructs within each domain are retained in a more parsimonious form. At the same time, some original content was reduced where items did not form stable, interpretable factors in this sample (e.g., Predictability and Job Satisfaction, as well as selected single-item indicators in the Conflicts/Offensive Behaviors domain), which may narrow coverage of those specific areas. We therefore explicitly document where content is reduced (S7 Table) and recommend that future studies test whether these constructs can be recovered through revised wording, additional items, or alternative model specifications in larger samples.

A further strength is that missing data were limited and handled in line with COPSOQ guidelines: respondents with more than 15% missing within a domain were excluded from analyses for that domain, and within-scale mean imputation was applied when less than 15% of items were missing so that all dimension scores could be expressed on the standard 0–100 metric. Multiple-imputation sensitivity analyses for ordinal items showed that the extracted factor structures, reliability indices, and the number of retained factors were highly robust to alternative ways of handling missing data. Tucker’s factor congruence coefficients between the main and MI-pooled EFAs were consistently high, and ordinal α and ω values changed only marginally with overlapping confidence intervals, supporting the stability of the findings.

Preliminary CFAs conducted separately for each domain provided additional support for the revised factor structures. Across domains, global fit indices were in the acceptable to excellent range (generally CFI/TLI ≥ 0.90, RMSEA ≤ 0.08, SRMR ≤ 0.08), and Fornell–Larcker and HTMT indices indicated satisfactory discriminant validity between most subscales. As expected, some limitations emerged for very short scales: Workplace Autonomy, Demands for Hiding Emotions, Emotional & Decision Demands, and Goal-Directed Self-Efficacy showed somewhat lower AVE or reliability indices, in line with their two- to four-item length and moderate standardized loadings. In the Health and Well-being domain, the Somatic Stress and Stress subscales displayed substantial overlap, with a high latent correlation and borderline discriminant validity, although HTMT values still remained at or below the commonly used 0.85 cut-off. Taken together, these patterns suggest that the revised Flemish COPSOQ III largely captures distinct, internally consistent constructs, but that some brief scales should be interpreted with more caution and may benefit from refinement in future work. Across all domains, we conducted preliminary CFAs as an initial check of the EFA-derived structures rather than definitive confirmation. Because these models relied on listwise-complete subsamples with modest Ns (approximately 220–242 out of 242) and reused the same dataset as the EFAs, the CFA results should be interpreted as tentative and in need of replication in larger, independent samples. For Personality, future research may also refine the GD items or test a higher-order self-efficacy factor if warranted.

Our results are broadly consistent with previous COPSOQ validation studies, which have also reported the need for contextual adaptation of specific scales while confirming the overall robustness of the COPSOQ framework. Various versions of COPSOQ II have been validated for specific groups (e.g., Australian school principals [24], Polish human services workers [14] and Egyptian oil and gas industry employees [25]), and adapted for national contexts (e.g., Peru [26], Hungary [27], Canada [28], France [29], and Germany [30]. Similarly, COPSOQ III, validated across different countries, often requires modifications to align with local contexts and professional groups [15]. For instance, Sahan et al. (2018) found that the Turkish version of COPSOQ III was a reliable and valid tool for measuring psychosocial risks, despite some dimensions, such as *Demands for Hiding Emotions*, showing lower reliability—similar to our findings [31]. Moreover, validation studies of COPSOQ III in German [32], Swedish [17], and Persian [33] populations reported satisfactory reliability and validity, confirming the correlation between work factors and outcome variables. The German adaptation of COPSOQ III, for example, involved modifications to meet both international standards and Germany’s unique workplace features [32]. The Norwegian validation of COPSOQ III, conducted in a sample of registered nurses, similarly identified deviations in the *Demands for Hiding Emotions* dimension, suggesting the need for scale simplification—paralleling our own findings [34]. A potential for item reduction was also highlighted in the Portuguese middle version of COPSOQ III [35].

This study also has several methodological and practical strengths. Psychometrically, it combines polychoric EFA, comprehensive reliability indices (including ω_total and ω_hierarchical), multiple-imputation sensitivity analyses, and preliminary CFA with convergent and discriminant validity tests, providing a nuanced evaluation of the Flemish COPSOQ III. Substantively, the revised set of subscales retains broad coverage of key psychosocial risk factors—demands, job contents, interpersonal relations, work–individual interface, social capital, conflicts, health and well-being, and personality—while remaining manageable in length and interpretable for practitioners. The findings suggest that the revised Flemish COPSOQ III can be used to support psychosocial risk assessment in healthcare settings, provided that users pay attention to the more tentative subscales and interpret them alongside the stronger dimensions.

### Limitations

This study has several limitations. First, it relied on a convenience sample drawn from three healthcare institutions, which may restrict the generalizability of the findings to the wider Flemish healthcare sector and to other occupational groups. In addition, the sample showed a substantial gender imbalance (79.8% women), and participation was uneven across sites, with most respondents drawn from the orthopedic rehabilitation facility (70.7%) compared with the two residential care centers (21.5% and 7.9%). These imbalances may introduce selection bias and context effects (e.g., site-specific working conditions) and therefore limit the generalizability of the revised structure. Future studies should replicate the findings using larger, more balanced samples and formally test measurement invariance across gender and workplace settings/sites. All data were collected cross-sectionally and based on self-report, which introduces the possibility of common-method and social desirability bias and precludes causal inferences about associations between psychosocial work factors and health outcomes.

Second, although the total sample size of 242 participants is acceptable for domain-level exploratory psychometric analysis, it remains modest relative to the overall length of the long COPSOQ III version (138 items). To keep the participant-to-item ratio within commonly recommended ranges, we estimated EFAs and CFAs separately within each domain rather than fitting one very large model including all 138 items, so that each analysis involved a more favourable ratio of cases to indicators. Even so, the effective sample sizes for the domain-specific CFAs were somewhat reduced by listwise deletion, and the same respondents contributed to both EFAs and CFAs. Recent simulation studies on CFA with ordinal Likert indicators emphasize that required sample sizes should be based on model complexity and communalities rather than simple per-item rules, and that for models with multiple factors and modest loadings several hundred cases are often needed for stable estimation and trustworthy fit indices [36,37]. In light of these recommendations, our total N of 242 and the absence of a split-sample EFA/CFA design mean that the current CFAs should be interpreted as preliminary internal checks of the revised COPSOQ III structure rather than definitive confirmatory evidence; replication in larger, independent samples with higher participant-to-item ratios is needed to verify the stability of the factor structures and model-fit indices.

Third, some of the brief subscales (e.g., Workplace Autonomy, Demands for Hiding Emotions, and Goal-Directed Self-Efficacy) showed somewhat lower AVE or reliability estimates compared with longer scales. These dimensions were retained because they cover theoretically important constructs and showed generally acceptable factor loadings, but their scores should be interpreted with caution, and future research may consider revising item wording, adding further items, or modelling higher-order factors to strengthen their measurement properties. The borderline discriminant validity between Somatic Stress and Stress also suggests substantial conceptual overlap, and alternative, more parsimonious factor structures for these symptom clusters may be worth exploring in future studies.

## Conclusions

In conclusion, this study provides preliminary evidence on internal consistency and construct-related validity, and proposes a contextually adapted structure of the extended Flemish COPSOQ III among healthcare workers, while also highlighting areas where the instrument can be improved. The revised factor structures capture theoretically meaningful aspects of the psychosocial work environment, and most subscales exhibit satisfactory internal consistency, convergent validity, and discriminant validity. At the same time, some brief scales—particularly Workplace Autonomy, Emotional & Decision Demands, Demands for Hiding Emotions, Goal-Directed Self-Efficacy, and the closely related Somatic Stress and Stress subscales—require cautious interpretation and may benefit from targeted refinement. Future research should replicate these findings in larger and more diverse samples, ideally using a priori, simulation-based sample size planning for WLSMV models (e.g., Monte Carlo studies implemented with R packages such as simsem) [38], examine measurement invariance across occupational and demographic groups, and explore shorter versions derived from the present long form. Such work will further clarify how COPSOQ III can best be used to monitor psychosocial risks and inform preventive interventions in healthcare and beyond.

## Supporting information

S1 Table Descriptive statistics and scale scoring. (DOCX)

S2 Table Item-level missingness and little’s MCAR test. (DOCX)

S3 Table Multiple-imputation (MI) Settings and EFA diagnostics. (DOCX)

S4 Table Tucker’s factor congruence. (DOCX)

S5 Table Reliability comparison: Main EFA vs MI-pooled EFA. (DOCX)

S6 Table Preliminary CFA model fit indices. (DOCX)

S7 Table Mapping of COPSOQ III dimensions to the extended flemish version. (DOCX)

S1 File The full questionnaire (Extended Flemish version of COPSOQ III, adapted for healthcare workers) is provided as Supporting Information. (PDF)

S2 File Inclusivity in global research questionnaire. (DOCX)
