# Supplementary material for: Streamlining psychosocial risk assessment: An exploratory adaptation of the COPSOQ III for Flemish healthcare workers
Source: PLoS One. 2026 Feb 5;21(2):e0342380. doi: 10.1371/journal.pone.0342380 (PMC12875473; doi:10.1371/journal.pone.0342380)
Supplement: S1 Table — (DOCX) [file pone.0342380.s001.docx]

Descriptive Statistics and Scale Scoring (0–100)

S1-1. Descriptive Statistics and Scale Scoring (0–100) for Demands at work

| **Scale** | **N total** | **N scored** | **N removed (>15% missing)** | **% removed** | **Missing cells (total)** | **Missing cells imputed*** | **Mean (raw)** | **SD (raw)** | **Min–max (raw)** | **Mean (0–100)** | **SD**  **(0–100)** | **Min–max (0–100)** | **Median**  **(0–100)** | **IQR (0–100)** |
| --- | --- | --- | --- | --- | --- | --- | --- | --- | --- | --- | --- | --- | --- | --- |
| Emotional & Decision Demands (ED) | 242 | 241 | 1 | 0.41 | 1 | 0 | 3.08 | 0.80 | 1.25–5.00 | 52.10 | 20.04 | 6.25–100.00 | 50.00 | 31.25 |
| Work pace (WP) | 242 | 239 | 3 | 1.24 | 3 | 0 | 2.82 | 0.80 | 1.00–5.00 | 45.57 | 19.99 | 0.00–100.00 | 41.67 | 25.00 |
| Quantitative Demands (QD) | 242 | 238 | 4 | 1.65 | 4 | 0 | 3.30 | 0.45 | 2.00–4.33 | 57.42 | 11.27 | 25.00–83.33 | 58.33 | 16.67 |
| Cognitive Demands (CD) | 242 | 238 | 4 | 1.65 | 4 | 0 | 2.29 | 0.65 | 1.00–4.00 | 32.32 | 16.20 | 0.00–75.00 | 33.33 | 16.67 |
| Demands for Hiding Emotions (HE) | 242 | 236 | 6 | 2.48 | 6 | 0 | 1.88 | 0.85 | 1.00–4.50 | 17.54 | 17.03 | 0.00–70.00 | 10.00 | 30.00 |
| Total domain score | 242 | 242 | 0 | 0.00 | 18 | 18 | 2.76 | 0.44 | 1.40–4.07 | 44.02 | 10.95 | 10.00–76.67 | 43.33 | 15.00 |

S1-2. Descriptive Statistics and Scale Scoring (0–100) for Work Organization and Job Contents

| **Scale** | **N total** | **N scored** | **N removed (>15% missing)** | **% removed** | **Missing cells (total)** | **Missing cells imputed*** | **Mean (raw)** | **SD (raw)** | **Min–max (raw)** | **Mean (0–100)** | **SD**  **(0–100)** | **Min–max**  **(0–100)** | **Median (0–100)** | **IQR (0–100)** |
| --- | --- | --- | --- | --- | --- | --- | --- | --- | --- | --- | --- | --- | --- | --- |
| Possibilities for development (PD) | 242 | 239 | 3 | 1.24 | 4 | 0 | 2.64 | 0.88 | 1.00–5.00 | 41.00 | 22.06 | 0–100 | 41.67 | 33.33 |
| Meaning of Work (MW) | 242 | 239 | 3 | 1.24 | 3 | 0 | 1.93 | 0.76 | 1.00–5.00 | 23.17 | 19.00 | 0–100 | 25.00 | 31.25 |
| Workplace Autonomy (WA) | 242 | 234 | 8 | 3.31 | 9 | 0 | 3.80 | 0.64 | 1.75–5.00 | 69.95 | 15.99 | 18.75–100 | 68.75 | 18.75 |
| Variation of Work (VA) | 242 | 240 | 2 | 0.83 | 2 | 0 | 2.69 | 0.92 | 1.00–5.00 | 42.29 | 23.02 | 0–100 | 37.50 | 37.5 |
| Influence at Work (IN) | 242 | 235 | 7 | 2.89 | 7 | 0 | 2.57 | 0.83 | 1.00–5.00 | 39.31 | 20.85 | 0–100 | 37.50 | 25.00 |
| Total domain score | 242 | 238 | 4 | 1.65 | 25 | 17 | 2.88 | 0.52 | 1.46–4.69 | 46.93 | 12.89 | 11.54–92.31 | 46.15 | 15.38 |

S1-3. Descriptive Statistics and Scale Scoring (0–100) for Interpersonal relations and leadership

| **Scale** | **N total** | **N scored** | **N removed (>15% missing)** | **% removed** | **Missing cells (total)** | **Missing cells imputed*** | **Mean (raw)** | **SD (raw)** | **Min–max (raw)** | **Mean**  **(0–100)** | **SD**  **(0–100)** | **Min–max (0–100)** | **Median (0–100)** | **IQR (0–100)** |
| --- | --- | --- | --- | --- | --- | --- | --- | --- | --- | --- | --- | --- | --- | --- |
| Quality of Leadership (QL) | 242 | 236 | 6 | 2.48 | 6 | 0 | 2.76 | 0.83 | 1.0–5.0 | 44.09 | 20.65 | 0–100.00 | 45.00 | 25.00 |
| Recognition (RE) | 242 | 239 | 3 | 1.24 | 4 | 0 | 2.32 | 0.82 | 1.0–5.0 | 33.02 | 20.47 | 0–100.00 | 25.00 | 25.00 |
| Sense of community at work (SW) | 242 | 237 | 5 | 2.07 | 14 | 0 | 1.99 | 0.71 | 1.0–4.0 | 33.05 | 23.57 | 0–100.00 | 33.33 | 22.22 |
| Role Clarity (CL) | 242 | 239 | 3 | 1.24 | 3 | 0 | 1.87 | 0.61 | 1.0–4.0 | 29.06 | 20.28 | 0–100.00 | 33.33 | 22.22 |
| Role & Task Conflict (RT) | 242 | 238 | 4 | 1.65 | 5 | 0 | 3.83 | 0.80 | 1.0–5.0 | 70.69 | 20.09 | 0–100.00 | 75.00 | 25.00 |
| Social support from colleagues (SC) | 242 | 236 | 6 | 2.48 | 10 | 0 | 2.50 | 0.76 | 1.0–5.0 | 37.54 | 18.95 | 0–100.00 | 33.33 | 25.00 |
| Total domain score | 242 | 239 | 3 | 1.24 | 42 | 27 | 2.57 | 0.46 | 1.4–4.0 | 39.26 | 11.61 | 10–75.00 | 38.75 | 15.00 |

S1-4. Descriptive Statistics and Scale Scoring (0–100) for Work-individual interface

| **Scale** | **N total** | **N scored** | **N removed (>15% missing)** | **% removed** | **Missing cells (total)** | **Missing cells imputed*** | **Mean (raw)** | **SD (raw)** | **Min–max (raw)** | **Mean (0–100)** | **SD**  **(0–100)** | **Min–max (0–100)** | **Median (0–100)** | **IQR**  **(0–100)** |
| --- | --- | --- | --- | --- | --- | --- | --- | --- | --- | --- | --- | --- | --- | --- |
| Commitment to the workplace (CW) | 242 | 238 | 4 | 1.65 | 4 | 0 | 3.70 | 0.78 | 1.20–5.00 | 67.50 | 19.52 | 5.00–100.00 | 70.00 | 25.00 |
| Work life conflict (WF) | 242 | 236 | 6 | 2.48 | 14 | 0 | 3.29 | 0.70 | 1.20–4.40 | 57.29 | 17.43 | 5.00–85.00 | 60.00 | 25.00 |
| Insecurity over working conditions (IW) | 242 | 241 | 1 | 0.41 | 4 | 0 | 3.79 | 0.94 | 1.00–5.00 | 69.74 | 23.62 | 0.00–100.00 | 75.00 | 31.25 |
| Work engagement (WE) | 242 | 240 | 2 | 0.83 | 6 | 0 | 5.44 | 1.25 | 1.00–7.00 | 73.98 | 20.91 | 0.00–100.00 | 77.78 | 27.78 |
| Insecurity over employment (JI) | 242 | 240 | 2 | 0.83 | 6 | 0 | 4.03 | 0.93 | 1.00–5.00 | 75.66 | 23.21 | 0.00–100.00 | 83.33 | 35.42 |
| Quality of work (QW) | 242 | 234 | 8 | 3.31 | 15 | 0 | 2.70 | 1.08 | 1.00–5.00 | 42.41 | 27.03 | 0.00–100.00 | 37.50 | 25.00 |
| Total domain score | 242 | 238 | 4 | 1.65 | 49 | 21 | 3.81 | 0.53 | 2.14–4.91 | 70.34 | 13.32 | 28.41–97.73 | 72.73 | 19.20 |

S1-5. Descriptive Statistics and Scale Scoring (0–100) for Social Capital

| **Scale** | **N total** | **N scored** | **N removed (>15% missing)** | **% removed** | **Missing cells (total)** | **Missing cells imputed** | **Mean (raw)** | **SD (raw)** | **Min–max (raw)** | **Mean**  **(0–100)** | **SD**  **(0–100)** | **Min–max**  **(0–100)** | **Median (0–100)** | **IQR**  **(0–100)** |
| --- | --- | --- | --- | --- | --- | --- | --- | --- | --- | --- | --- | --- | --- | --- |
| Organizational Justice (JU) | 242 | 239 | 3 | 1.24 | 3 | 0 | 2.78 | 0.75 | 1.0–5.0 | 44.58 | 18.73 | 0.00–100.00 | 45 | 22.5 |
| Horizontal Trust (TE) | 242 | 236 | 6 | 2.48 | 10 | 0 | 2.15 | 0.88 | 1.0–5.0 | 28.81 | 22.1 | 0.00–100.00 | 25 | 33.33 |
| Vertical Trust (TM) | 242 | 240 | 2 | 0.83 | 4 | 0 | 2.06 | 0.67 | 1.0–4.5 | 26.46 | 16.86 | 0.00–87.50 | 25 | 25.00 |
| Total domain score | 242 | 239 | 3 | 1.24 | 17 | 3 | 2.45 | 0.64 | 1.0–4.4 | 36.15 | 16.08 | 0.00–85.00 | 35 | 20.00 |

S1-6. Descriptive Statistics and Scale Scoring (0–100) for Conflicts and Offensive Behaviors

| **Scale** | **N total** | **N scored** | **N removed (≥15% missing)** | **% removed** | **Missing cells (total)** | **Missing cells imputed** | **Mean (raw)** | **SD (raw)** | **Min–max (raw)** | **Mean (0–100)** | **SD**  **(0–100)** | **Min–max**  **(0–100)** | **Median**  **(0–100)** | **IQR**  **(0–100)** |
| --- | --- | --- | --- | --- | --- | --- | --- | --- | --- | --- | --- | --- | --- | --- |
| [Workplace Behavioral Transgression (WBT)](https://link.springer.com/article/10.1007/s10869-019-09622-1) | 242 | 242 | 0 | 0 | 0 | 0 | 4.75 | 0.56 | 1.0–5.0 | 93.73 | 14.09 | 0.00–100.00 | 100 | 8.33 |
| Violence and Harassment (VH) | 242 | 242 | 0 | 0 | 0 | 0 | 4.81 | 0.50 | 1.3–5.0 | 95.14 | 12.58 | 8.33–100.00 | 100 | 0.00 |
| Total domain score | 242 | 242 | 0 | 0 | 0 | 0 | 4.78 | 0.40 | 1.7–5.0 | 94.44 | 10.06 | 16.67–100.00 | 100 | 8.33 |

S1-7. Descriptive Statistics and Scale Scoring (0–100) for Health and Well-being

| **Scale** | **N total** | **N scored** | **N removed (≥15% missing)** | **% removed** | **Missing cells (total)** | **Missing cells imputed** | **Mean (raw)** | **SD (raw)** | **Min–max (raw)** | **Mean**  **(0–100)** | **SD**  **(0–100)** | **Min–max**  **(0–100)** | **Median (0–100)** | **IQR**  **(0–100)** |
| --- | --- | --- | --- | --- | --- | --- | --- | --- | --- | --- | --- | --- | --- | --- |
| Cognitive Well-being Assessment (CWA) | 242 | 240 | 2 | 0.83 | 2 | 0 | 4.26 | 0.68 | 1.8–5.0 | 81.60 | 17.09 | 20.00–100.00 | 85 | 20.00 |
| Burnout (BO) | 242 | 238 | 4 | 1.65 | 4 | 0 | 3.45 | 0.92 | 1.0–5.0 | 61.31 | 23.06 | 0.00–100.00 | 66.67 | 25.00 |
| Sleeping Troubles (SL) | 242 | 240 | 2 | 0.83 | 2 | 0 | 3.68 | 0.93 | 1.0–5.0 | 66.95 | 23.31 | 0.00–100.00 | 75.00 | 31.25 |
| Somatic Stress (SO) | 242 | 239 | 3 | 1.24 | 3 | 0 | 4.19 | 0.81 | 1.0–5.0 | 79.71 | 20.36 | 0.00–100.00 | 83.33 | 25.00 |
| Stress (ST) | 242 | 242 | 0 | 0.00 | 0 | 0 | 3.63 | 0.90 | 1.0–5.0 | 65.86 | 22.60 | 0.00–100.00 | 75.00 | 25.00 |
| Total domain score | 242 | 242 | 0 | 0.00 | 11 | 11 | 3.90 | 0.66 | 1.24–5.0 | 72.43 | 16.51 | 5.88–100.00 | 75.00 | 22.06 |

S1-8. Descriptive Statistics and Scale Scoring (0–100) for Personality

| **Scale** | **N total** | **N scored** | **N removed (≥15% missing)** | **% removed** | **Missing cells (total)** | **Missing cells imputed** | **Mean (raw)** | **SD (raw)** | **Min–max (raw)** | **Mean**  **(0–100)** | **SD**  **(0–100)** | **Min–max**  **(0–100)** | **Median (0–100)** | **IQR**  **(0–100)** |
| --- | --- | --- | --- | --- | --- | --- | --- | --- | --- | --- | --- | --- | --- | --- |
| Problem-Solving Self-Efficacy (PS) | 242 | 240 | 2 | 0.80 | 2 | 0 | 2.07 | 0.51 | 1.0–4.0 | 35.80 | 16.89 | 0.00–100.00 | 33.33 | 10.42 |
| Goal-Directed Self-Efficacy (GD) | 242 | 241 | 1 | 0.40 | 1 | 0 | 2.44 | 0.62 | 1.0–4.0 | 47.99 | 20.62 | 0.00–100.00 | 50.00 | 33.33 |
| Total domain score | 242 | 239 | 3 | 1.20 | 3 | 0 | 2.20 | 0.47 | 1.0–3.8 | 29.88 | 11.82 | 0.00–70.83 | 29.17 | 12.50 |
