# Supplementary material for: Streamlining psychosocial risk assessment: An exploratory adaptation of the COPSOQ III for Flemish healthcare workers
Source: PLoS One. 2026 Feb 5;21(2):e0342380. doi: 10.1371/journal.pone.0342380 (PMC12875473; doi:10.1371/journal.pone.0342380)
Supplement: S2 Table — (DOCX) [file pone.0342380.s002.docx]

S2_1. Item-level Missingness and Little’s MCAR Test (Demands at work) (N = 242)

| **Item** | **QD1** | **QD2** | **QD4** | **WP1** | **WP2** | **WP3** | **CD1** | **CD2** | **CD3** | **CD4** | **ED1** | **ED2** | **ED3** | **HE1** | **HE3** |
| --- | --- | --- | --- | --- | --- | --- | --- | --- | --- | --- | --- | --- | --- | --- | --- |
| **Missing %** | 0.41 | 0.83 | 0.00 | 0.83 | 0.00 | 0.41 | 0.83 | 0.83 | 0.00 | 0.00 | 0.00 | 0.00 | 0.41 | 2.48 | 0.00 |

Note: χ²(202) = 292.35, p < .001, missing patterns = 13. Little’s MCAR test was significant, indicating that the strict MCAR assumption did not hold. We proceeded under MAR assumptions and used multiple imputation for ordinal items.

S2_2. Item-level missingness and Little’s MCAR test (Work organization and job contents) (N = 242)

| **Item** | **MW1** | **MW2** | **PD1** | **PD2** | **PD3** | **IN1** | **IN2** | **IN3** | **IN4** | **IN5** | **IN6** | **CT1** | **CT2** | **CT3** | **CT4** | **CT5** | **VA1** | **VA2** |
| --- | --- | --- | --- | --- | --- | --- | --- | --- | --- | --- | --- | --- | --- | --- | --- | --- | --- | --- |
| **Missing (%)** | 1.24 | 0.00 | 0.00 | 0.41 | 1.24 | 0.00 | 1.65 | 0.83 | 1.65 | 1.24 | 1.24 | 0.00 | 0.41 | 0.00 | 0.00 | 0.41 | 0.83 | 0.00 |

Note: χ²(217) = 241.98, p = .118 > 0.05, missing-data patterns = 14. Little’s MCAR test was non-significant, indicating that the missingness pattern was approximately consistent with MCAR (and therefore compatible with standard MAR-based methods). Then we used multiple imputation for ordinal items.

S2_3. Item-level missingness and Little’s MCAR test (Interpersonal relations and leadership) (N = 242)

| **Item** | **PR1** | **PR2** | **RE1** | **RE3** | **RE2** | **QL1** | **QL2** | **QL3** | **QL4** | **CL1** | **CL2** | **CL3** |
| --- | --- | --- | --- | --- | --- | --- | --- | --- | --- | --- | --- | --- |
| **Missing %** | 0.00 | 0.00 | 1.24 | 0.00 | 0.41 | 0.00 | 0.00 | 1.65 | 0.00 | 1.24 | 0.00 | 0.00 |
| **Item** | **CO2** | **CO3** | **IT1** | **SS1** | **SS2** | **SS3** | **SC1** | **SC2** | **SC3** | **SW1** | **SW2** | **SW3** |
| **Missing %** | 0.41 | 0.41 | 1.24 | 0.83 | 0.83 | 0.83 | 1.24 | 0.83 | 2.07 | 2.07 | 2.07 | 1.65 |

Note: χ²(391) = 410.46, p = .239 > 0.05, distinct missing-data patterns = 19. Little’s MCAR test was non-significant, indicating that the missingness pattern was approximately consistent with MCAR (and therefore compatible with standard MAR-based methods). Then we used multiple imputation for ordinal items.

S2_4. Item-level missingness and Little’s MCAR test (Work-Individual Interface) (N = 242)

| **Item** | **JI1** | **JI2** | **JI3** | **IW1** | **IW2** | **IW3** | **IW4** | **IW5** | **QW1** | **QW2** | **WF1** | **WF2** | **WF3** | **WF5** |
| --- | --- | --- | --- | --- | --- | --- | --- | --- | --- | --- | --- | --- | --- | --- |
| **Missing (%)** | 0.83 | 0.83 | 0.83 | 0.41 | 0.41 | 0.41 | 0.41 | 1.24 | 3.31 | 2.89 | 1.65 | 0.83 | 1.24 | 1.24 |
| **Item** | **WF4** | **CW1** | **CW2** | **CW3** | **CW4** | **CW5** | **WE1** | **WE2** | **WE3** | **JS1** | **JS2** | **JS3** | **JS4** | **JS5** |
| **Missing (%)** | 0.83 | 0.41 | 0.00 | 0.00 | 1.24 | 0.00 | 0.83 | 0.83 | 0.83 | 4.96 | 2.48 | 2.07 | 1.24 | 1.24 |

Note: χ²(527) = 588.95, p = .032 < 0.05, missing-data patterns = 22. Little’s MCAR test was statistically significant, suggesting the data deviate slightly from strict MCAR. Given the small amount of missingness, it is reasonable to proceeded under MAR mechanism and to use multiple imputation as a sensitivity check.

S2_5. Item-level missingness and Little’s MCAR test (Social Capital) (N = 242)

| **Item** | **JU1** | **JU3** | **JU2** | **JU4** | **TE1** | **TE2** | **TE3** | **TM1** | **TM2** | **TM3** | **TM4** |
| --- | --- | --- | --- | --- | --- | --- | --- | --- | --- | --- | --- |
| **Missing %** | 0.41 | 0 | 0 | 0 | 1.24 | 1.24 | 1.24 | 0.83 | 0.83 | 1.65 | 0.83 |

Note: χ²(32) = 40.26, p = .15 > 0.05, distinct missing-data patterns = 5. Little’s MCAR test was non-significant, suggesting an approximately MCAR pattern compatible with standard MAR-based methods and multiple imputation.

S2_6. Item-level missingness and Little’s MCAR test (Conflicts and Offensive Behaviors( (N = 242)

| **Item** | **BU1** | **BU2** | **GS1** | **CQ1** | **UT1** | **HSM1** | **SH1** | **TV1** | **PV1** |
| --- | --- | --- | --- | --- | --- | --- | --- | --- | --- |
| **Missing %** | 0 | 0 | 0 | 0 | 0 | 0 | 0 | 0 | 0 |

Note: All nine items had complete data (0% missing). As a result, Little’s MCAR test was degenerate (df = 0), and missingness was not a concern.

S2_7. Item-level missingness and Little’s MCAR test (Health and Well-being) (N = 242)

| **Item** | **BO1** | **BO3** | **BO2** | **BO4** | **CS1** | **CS2** | **CS3** | **CS4** | **DS1** | **DS2** | **DS3** | **DS4** |
| --- | --- | --- | --- | --- | --- | --- | --- | --- | --- | --- | --- | --- |
| **Missing %** | 0.41 | 0.83 | 0.83 | 0.41 | 0.41 | 0.00 | 0.00 | 0.41 | 0.41 | 0.00 | 0.00 | 0.00 |
| **Item** | **GH1** | **SL1** | **SL2** | **SL3** | **SL4** | **SO1** | **SO2** | **SO3** | **SO4** | **ST1** | **ST2** | **ST3** |
| **Missing %** | 0.41 | 0.00 | 0.00 | 0.83 | 0.00 | 0.41 | 0.41 | 0.41 | 0.00 | 0.00 | 0.00 | 0.00 |

Note: χ²(275) = 282.68, p = .36 > 0.5, missing-data patterns =13, Little’s MCAR test was non-significant, suggesting an approximately MCAR pattern compatible with standard MAR-based methods and multiple imputation.

S2_8. Item-level missingness and Little’s MCAR test (Personality) (N = 242)

| **Item** | **SE1** | **SE2** | **SE3** | **SE4** | **SE5** | **SE6** |
| --- | --- | --- | --- | --- | --- | --- |
| **Missing (%)** | 0.00 | 0.41 | 0.00 | 0.83 | 0.00 | 0.00 |

Note: Item-level missingness was negligible (<1%) across all SE items. Little’s MCAR test was significant (p < .05), indicating that strict MCAR is unlikely; therefore, we proceeded under MAR assumptions and assessed robustness via multiple-imputation sensitivity analyses.
