# Supplementary material for: Streamlining psychosocial risk assessment: An exploratory adaptation of the COPSOQ III for Flemish healthcare workers
Source: PLoS One. 2026 Feb 5;21(2):e0342380. doi: 10.1371/journal.pone.0342380 (PMC12875473; doi:10.1371/journal.pone.0342380)
Supplement: S3 Table — (DOCX) [file pone.0342380.s003.docx]

**Multiple-imputation (MI) Settings and EFA Diagnostics**

S3_1. Multiple-imputation settings and pooled EFA diagnostics for Demands at work

| **Feature** | **Items in domain** | **Number of imputations**  **(m)** | **Parallel analysis on**  **MI-pooled correlation** | **KMO**  **(MI pooled)** | **Bartlett’s test**  **(MI pooled)** |
| --- | --- | --- | --- | --- | --- |
| **Value / description** | 15 Likert-type items  (QD1–QD2, QD4, WP1–WP3, CD1–CD4, ED1, ED2, ED3, HE1, HE3) | 65 | Parallel analysis  (MI-pooled R): 5 | 0.77 (middling) | χ²(105) = 1271.11,  p < .001 |

S3_2. Multiple-imputation settings and pooled EFA diagnostics for Work Organization and Job Contents

| **Feature** | **Items in domain** | **Number of imputations**  **(m)** | **Parallel analysis on**  **MI-pooled correlation** | **KMO**  **(MI pooled)** | **Bartlett’s test**  **(MI pooled)** |
| --- | --- | --- | --- | --- | --- |
| **Value / description** | 13 Likert-type items  (MW1–MW2, PD1–PD3, IN2–IN6, CT4, VA1, VA2) | 85 | Parallel analysis  (MI-pooled R): 5 | 0.78 (middling) | χ²(78) = 1141.60,  p < .001 |

S3_3. Multiple-imputation settings and pooled EFA diagnostics for Interpersonal Relations and Leadership

| **Feature** | **Items in domain** | **Number of imputations**  **(m)** | **Parallel analysis on**  **MI-pooled correlation** | **KMO**  **(MI pooled)** | **Bartlett’s test**  **(MI pooled)** |
| --- | --- | --- | --- | --- | --- |
| **Value / description** | 20 Likert-type items  (RE1, RE3, RE2, QL1, QL2, QL3, QL4, CL1, CL2, CL3, CO2, CO3, IT1, SS3, SC1, SC2, SC3, SW1, SW2, SW3) | 134 | Parallel analysis  (MI-pooled R): 6 | 0.88 (meritorious overall sampling adequacy) | χ²(190) = 3025.93,  p < .001 |

S3_4. Multiple-imputation settings and pooled EFA diagnostics for Work-Individual Interface

| **Feature** | **Items in domain** | **Number of imputations**  **(m)** | **Parallel analysis on**  **MI-pooled correlation** | **KMO**  **(MI pooled)** | **Bartlett’s test**  **(MI pooled)** |
| --- | --- | --- | --- | --- | --- |
| **Value / description** | 22 Likert-type items  (JI1–JI3, IW1–IW4, QW1–QW2, WF1, WF2–WF3, WF5, WF4, CW1–CW5, WE1–WE3) | 57 | Parallel analysis  (MI-pooled R): 6 | 0.82 (meritorious overall sampling adequacy) | χ²(231) = 3053.41,  p < .001 |

S3_5. Multiple-imputation settings and pooled EFA diagnostics for Social Capital

| **Feature** | **Items in domain** | **Number of imputations**  **(m)** | **Parallel analysis on**  **MI-pooled correlation** | **KMO**  **(MI pooled)** | **Bartlett’s test**  **(MI pooled)** |
| --- | --- | --- | --- | --- | --- |
| **Value / description** | 10 Likert-type items  (JU1, JU3, JU2, JU4, TE1, TE2, TM1, TM2, TM3, TM4) | 26 | Parallel analysis  (MI-pooled R): 3 | 0.87 (meritorious overall sampling adequacy) | χ²(45) = 1196.40,  p < .001 |

S3_6. Multiple-imputation settings and pooled EFA diagnostics for Conflicts and Offensive Behaviors

| **Feature** | **Items in domain** | **Number of imputations (m)** | **Parallel analysis on MI-pooled correlation** | **KMO**  **(MI pooled)** | **Bartlett’s test**  **(MI pooled)** |
| --- | --- | --- | --- | --- | --- |
| **Value / description** | 6 Likert-type items  (BU1, BU2, UT1, SH1, TV1, PV1) | 3 | Parallel analysis  (MI-pooled R): 2 | 0.66 (mediocre overall sampling adequacy) | χ²(15) = 493.63,  p < .001 |

S3_6. Multiple-imputation settings and pooled EFA diagnostics for Health and Well-being

| **Feature** | **Items in domain** | **Number of imputations**  **(m)** | **Parallel analysis on**  **MI-pooled correlation** | **KMO**  **(MI pooled)** | **Bartlett’s test**  **(MI pooled)** |
| --- | --- | --- | --- | --- | --- |
| **Value / description** | 17 Likert-type items  (BO1, BO2, BO4, CS1–CS4, DS3, SL1–SL4, SO1–SO3, ST2–ST3) | 34 | Parallel analysis  (MI-pooled R): 5 | 0.91 (marvelous overall sampling adequacy) | χ²(136) = 2699.14,  p < .001 |

S3_6. Multiple-imputation settings and pooled EFA diagnostics for Personality

| **Feature** | **Items in domain** | **Number of imputations**  **(m)** | **Parallel analysis on**  **MI-pooled correlation** | **KMO**  **(MI pooled)** | **Bartlett’s test**  **(MI pooled)** |
| --- | --- | --- | --- | --- | --- |
| **Value / description** | 6 Likert-type items  (SE1–SE6) | 6 | Parallel analysis  (MI-pooled R): 2 | 0.81 (meritorious overall sampling adequacy) | χ²(15) = 378.50, p < .001 |
