# Supplementary material for: Streamlining psychosocial risk assessment: An exploratory adaptation of the COPSOQ III for Flemish healthcare workers
Source: PLoS One. 2026 Feb 5;21(2):e0342380. doi: 10.1371/journal.pone.0342380 (PMC12875473; doi:10.1371/journal.pone.0342380)
Supplement: S4 Table — (DOCX) [file pone.0342380.s004.docx]

**Factor Congruence**

S4_1. Tucker’s factor congruence between main polychoric EFA and MI-pooled EFA (Demands at work)

| **Factor label** | **Emotional & Decision Demands**  **(ED)** | **Quantitative Demands**  **(QD)** | **Work Pace (WP)** | **Cognitive Demands (CD)** | **Demands for Hiding Emotions**  **(HE)** |
| --- | --- | --- | --- | --- | --- |
| **Main factor ↔ MI factor** | PA2 ↔ PA2 | PA1 ↔ PA1 | PA3 ↔ PA5 | PA4 ↔ PA4 | PA5 ↔ PA3 |
| **Congruence** | 1.00 | 1.00 | 1.00 | 0.99 | 1.00 |

S4_2. Tucker’s factor congruence between main polychoric EFA and MI-pooled EFA (Work organization)

| **Factor label** | **Possibilities for Development (PD)** | **Meaning of Work (MW)** | **Workplace Autonomy (WA)** | **Variation of Work (VA)** | **Influence at Work**  **(IN)** |
| --- | --- | --- | --- | --- | --- |
| **Main factor ↔ MI factor** | PA1 ↔ PA1 | PA5 ↔ PA3 | PA2 ↔ PA2 | PA4 ↔ PA4 | PA3 ↔ PA5 |
| **Congruence** | 1.00 | 1.00 | 1.00 | 1.00 | 1.00 |

S4_3. Tucker’s factor congruence between main polychoric EFA and MI-pooled EFA (Interpersonal Relations and Leadership)

| **Factor label** | **Quality of Leadership (QL)** | **Recognition**  **(RE)** | **Sense of Community at Work**  **(SW)** | **Role Clarity (CL)** | **Role & Task Conflict**  **(RT)** | **Social Support from Colleagues**  **(SC)** |
| --- | --- | --- | --- | --- | --- | --- |
| **Main factor ↔ MI factor** | PA1 ↔ PA5 | PA6 ↔ PA1 | PA2 ↔ PA2 | PA4 ↔ PA4 | PA3 ↔ PA3 | PA5 ↔ PA6 |
| **Congruence** | 1.00 | 0.99 | 0.99 | 0.99 | 1.00 | 0.99 |

S4_4. Tucker’s factor congruence between main polychoric EFA and MI-pooled EFA (Work-Individual Interface)

| **Factor label** | **Commitment to the Workplace (CW)** | **Work-Life Conflict**  **(WF)** | **Insecurity Over Working Conditions**  **(IW)** | **Work Engagement**  **(WE)** | **Insecurity Over Employment**  **(JI)** | **Quality of Work (QW)** |
| --- | --- | --- | --- | --- | --- | --- |
| **Main factor ↔ MI factor** | PA1 ↔ PA1 | PA3 ↔ PA4 | PA2 ↔ PA2 | PA5 ↔ PA5 | PA6 ↔ PA6 | PA4 ↔ PA3 |
| **Congruence** | 1.00 | 1.00 | 1.00 | 1.00 | 1.00 | 1.00 |

S4_5. Tucker’s factor congruence between main polychoric EFA and MI-pooled EFA (Social Capital)

| **Factor label** | **Organizational Justice**  **(JU)** | **Horizontal Trust**  **(TE)** | **Vertical Trust**  **(TM)** |
| --- | --- | --- | --- |
| **Main factor → MI factor** | PA1 → PA1 | PA2 → PA2 | PA3 → PA3 |
| **Congruence** | 1.00 | 1.00 | 0.99 |

S4_6. Tucker’s factor congruence between main polychoric EFA and MI-pooled EFA (Conflicts and Offensive Behaviors)

| **Factor label** | **Workplace Behavioral Transgression**  **(WBT)** | **Violence and harassment**  **(VH)** |
| --- | --- | --- |
| **Main factor ↔ MI factor** | PA1 ↔ PA1 | PA2 ↔ PA2 |
| **Congruence** | 0.99 | 0.98 |

S4_7. Tucker’s factor congruence between main polychoric EFA and MI-pooled EFA (Health and Well-being)

| **Factor label** | **Cognitive Well-being Assessment**  **(CWA)** | **Burnout**  **(BO)** | **Sleeping Troubles**  **(SL)** | **Somatic Stress**  **(SO)** | **Stress**  **(ST)** |
| --- | --- | --- | --- | --- | --- |
| **Main factor ↔ MI factor** | PA3 ↔ PA3 | PA1 ↔ PA1 | PA2 ↔ PA2 | PA4 ↔ PA4 | PA5 ↔ PA5 |
| **Congruence** | 1.00 | 0.99 | 1.00 | 0.98 | 0.97 |

S4_8. Tucker’s factor congruence between main polychoric EFA and MI-pooled EFA (Personality)

| **Factor label** | **Problem-Solving Self-Efficacy (PS)** | **Goal-Directed Self-Efficacy (GD)** |
| --- | --- | --- |
| **Main factor ↔ MI factor** | PA1 ↔ PA1 | PA2 ↔ PA2 |
| **Congruence** | 1.00 | 0.99 |
