# Supplementary material for: Streamlining psychosocial risk assessment: An exploratory adaptation of the COPSOQ III for Flemish healthcare workers
Source: PLoS One. 2026 Feb 5;21(2):e0342380. doi: 10.1371/journal.pone.0342380 (PMC12875473; doi:10.1371/journal.pone.0342380)
Supplement: S5 Table — (DOCX) [file pone.0342380.s005.docx]

**Reliability Comparison: Main EFA vs MI-pooled EFA^[[1]](#footnote-1)^**

S5_1. Comparison of reliability indices from main EFA vs MI-pooled EFA (Demands at Work)

| **Subscale** | **Emotional & Decision Demands**  **(ED)** | **Quantitative Demands**  **(QD)** | **Work Pace (WP)** | **Cognitive Demands**  **(CD)** | **Demands for Hiding Emotions**  **(HE)** | **Overall**  **Demands at Work** |
| --- | --- | --- | --- | --- | --- | --- |
| **α_main (95% CI)** | 0.79 [0.74–0.83] | 0.85 [0.80–0.89] | 0.86 [0.83–0.90] | 0.71 [0.61–0.77] | 0.58 [0.43–0.69] | 0.82 [0.77–0.85] |
| **α_MI (95% CI)** | 0.76 [0.71–0.81] | 0.80 [0.75–0.84] | 0.82 [0.78–0.86] | 0.66 [0.59–0.73] | 0.48 [0.35–0.61] | 0.80 [0.77–0.84] |
| **ω_total, main (95% CI)** | 0.80 [0.75–0.84] | 0.85 [0.80–0.89] | 0.87 [0.84–0.90] | 0.74 [0.66–0.79] | 0.58 [0.42–0.70] | 0.90 [0.87–0.92] |
| **ω_total, MI (point)** | 0.77 | 0.8 | 0.83 | 0.68 | 0.48 | 0.89 |

S5_2. Comparison of reliability indices from main EFA vs MI-pooled EFA (Work Organization and Job Contents)

| **Subscale** | **Possibilities for Development (PD)** | **Meaning of Work**  **(MW)** | **Workplace Autonomy**  **(WA)** | **Variation of Work (VA)** | **Influence at Work (IN)** | **Overall Work Organization** |
| --- | --- | --- | --- | --- | --- | --- |
| **α_main (95% CI)** | 0.90 [0.86–0.92] | 0.92 [0.89–0.96] | 0.72 [0.65–0.78] | 0.79 [0.69–0.86] | 0.75 [0.66–0.83] | 0.83 [0.77–0.86] |
| **α_MI (95% CI)** | 0.87 [0.84–0.90] | 0.87 [0.84–0.90] | 0.65 [0.58–0.72] | 0.75 [0.68–0.81] | 0.69 [0.61–0.77] | 0.81 [0.77–0.84] |
| **ω_total, main (95% CI)** | 0.90 [0.87–0.93] | 0.92 [0.88–0.96] | 0.73 [0.65–0.79] | 0.79 [0.70–0.86] | 0.75 [0.65–0.83] | 0.92 [0.87–0.94] |
| **ω_total, MI (point)** | 0.87 | 0.87 | 0.66 | 0.75 | 0.69 | 0.89 |

S5_3. Comparison of reliability indices from main EFA vs MI-pooled EFA (Interpersonal Relations and Leadership)

| **Subscale** | **Quality of Leadership (QL)** | **Recognition (RE)** | **Sense of Community at Work**  **(SW)** | **Role Clarity (CL)** | **Role & Task Conflict**  **(RT)** | **Social Support from Colleagues (SC)** | **Overall Interpersonal Relations** |
| --- | --- | --- | --- | --- | --- | --- | --- |
| **α_main (95% CI)** | 0.88 [0.84–0.90] | 0.92 [0.77–0.94] | 0.93 [0.91–0.95] | 0.89 [0.85–0.92] | 0.82 [0.77–0.86] | 0.76 [0.68–0.80] | 0.88 [0.85–0.89] |
| **α_MI (95% CI)** | 0.87 [0.84–0.90] | 0.93 [0.92–0.95] | 0.88 [0.85–0.90] | 0.83 [0.79–0.86] | 0.79 [0.74–0.84] | 0.76 [0.71–0.81] | 0.86 [0.84–0.88] |
| **ω_total, main (95% CI)** | 0.89 [0.85–0.90] | 0.92 [0.90–0.94] | 0.93 [0.91–0.96] | 0.89 [0.85–0.93] | 0.82 [0.76–0.86] | 0.80 [0.75–0.84] | 0.96 [0.96–0.97] |
| **ω_total, MI (point)** | 0.88 | 0.93 | 0.88 | 0.83 | 0.79 | 0.79 | 0.94 |

S5_4. Comparison of reliability indices from main EFA vs MI-pooled EFA (Work-Individual Interface)

| **Subscale** | **Commitment to the Workplace (CW)** | **Work-Life Conflict (WF)** | **Insecurity Over Working Conditions (IW)** | **Work Engagement (WE)** | **Insecurity Over Employment (JI)** | **Quality of Work (QW)** | **Overall Work-Individual Interface** |
| --- | --- | --- | --- | --- | --- | --- | --- |
| **α_main (95% CI)** | 0.90 [0.87–0.92] | 0.84 [0.79–0.87] | 0.88 [0.84–0.91] | 0.94 [0.92–0.96] | 0.83 [0.77–0.87] | 0.93 [0.89–0.96] | 0.87 [0.85–0.89] |
| **α_MI (95% CI)** | 0.87 [0.85–0.90] | 0.81 [0.77–0.85] | 0.83 [0.80–0.87] | 0.91 [0.89–0.93] | 0.75 [0.70–0.81] | 0.92 [0.89–0.94] | 0.85 [0.82–0.88] |
| **ω_total, main (95% CI)** | 0.90 [0.88–0.92] | 0.85 [0.81–0.88] | 0.88 [0.84–0.91] | 0.94 [0.92–0.96] | 0.83 [0.78–0.88] | 0.93 [0.90–0.96] | 0.95 [0.94–0.96] |
| **ω_total, MI (point)** | 0.88 | 0.82 | 0.84 | 0.91 | 0.76 | 0.92 | 0.93 |

S5_5. Comparison of reliability indices from main EFA vs MI-pooled EFA (Social Capital)

| **Subscale** | **Organizational Justice (JU)** | **Horizontal Trust (TE)** | **Vertical Trust (TM)** | **Overall Social Capital** |
| --- | --- | --- | --- | --- |
| **α_main (95% CI)** | 0.90 [0.87–0.92] | 0.84 [0.79–0.89] | 0.81 [0.73–0.88] | 0.89 [0.86–0.91] |
| **α_MI (95% CI)** | 0.87 [0.85–0.90] | 0.80 [0.76–0.85] | 0.74 [0.67–0.80] | 0.88 [0.86–0.90] |
| **ω_total, main (95% CI)** | 0.90 [0.87–0.92] | 0.86 [0.82–0.90] | 0.81 [0.73–0.87] | 0.93 [0.91–0.94] |
| **ω_total, MI (point)** | 0.87 | 0.82 | 0.74 | 0.92 |

S5_6. Comparison of reliability indices from main EFA vs MI-pooled EFA (Conflicts and Offensive Behaviors)

| **Subscale** | [**Workplace Behavioral Transgression (WBT)**](https://link.springer.com/article/10.1007/s10869-019-09622-1) | **Violence and Harassment (VH)** | **Overall Conflicts and Offensive Behaviors** |
| --- | --- | --- | --- |
| **α_main (95% CI)** | 0.92 [0.86–0.95] | 0.86 [0.74–0.93] | 0.79 [0.71–0.87] |
| **α_MI (95% CI)** | 0.81 [0.76–0.85] | 0.77 [0.71–0.82] | 0.70 [0.64–0.76] |
| **ω_total, main (95% CI)** | 0.92 [0.86–0.95] | 0.86 [0.79–0.93] | 0.91 [0.87–0.95] |
| **ω_total, MI (point)** | 0.81 | 0.79 | 0.83 |

S5_7. Comparison of reliability indices from main EFA vs MI-pooled EFA (Health and Well-being)

| **Subscale** | **Cognitive Well-being Assessment**  **(CWA)** | **Burnout**  **(BO)** | **Sleeping Troubles (SL)** | **Somatic Stress (SO)** | **Stress**  **(ST)** | **Overall Health & Well-being** |
| --- | --- | --- | --- | --- | --- | --- |
| **α_main (95% CI)** | 0.89 [0.87–0.92] | 0.95 [0.93–0.97] | 0.89 [0.86–0.92] | 0.79 [0.72–0.84] | 0.92 [0.88–0.95] | 0.94 [0.92–0.95] |
| **α_MI (95% CI)** | 0.85 [0.82–0.88] | 0.92 [0.90–0.94] | 0.88 [0.85–0.91] | 0.74 [0.69–0.80] | 0.88 [0.85–0.91] | 0.93 [0.91–0.94] |
| **ω_total, main (95% CI)** | 0.90 [0.87–0.92] | 0.95 [0.94–0.97] | 0.90 [0.87–0.92] | 0.79 [0.71–0.84] | 0.92 [0.88–0.95] | 0.97 [0.96–0.97] |
| **ω_total, MI (point)** | 0.85 | 0.92 | 0.89 | 0.74 | 0.88 | 0.96 |

S5_8. Comparison of reliability indices from main EFA vs MI-pooled EFA (Personality)

| **Subscale** | **Problem-Solving Self-Efficacy**  **(PS)** | **Goal-Directed Self-Efficacy**  **(GD)** | **Overall Personality** |
| --- | --- | --- | --- |
| **α_main (95% CI)** | 0.84 [0.79–0.88] | 0.63 [0.49–0.75] | 0.83 [0.78–0.87] |
| **α_MI (95% CI)** | 0.79 [0.74–0.83] | 0.55 [0.43–0.66] | 0.78 [0.74–0.83] |
| **ω_total, main (95% CI)** | 0.85 [0.80–0.88] | 0.63 [0.45–0.75] | 0.86 [0.83–0.90] |
| **ω_total, MI (point)** | 0.79 | 0.55 | 0.81 |

1. MI results are reported as pooled point estimates; confidence intervals were not computed for MI because pooling uncertainty for non-linear reliability coefficients is not straightforward and m differed across domains. Conclusions are based on the close agreement between MI point estimates and the main-analysis estimates with 95% CIs. [↑](#footnote-ref-1)
