# Supplementary material for: Streamlining psychosocial risk assessment: An exploratory adaptation of the COPSOQ III for Flemish healthcare workers
Source: PLoS One. 2026 Feb 5;21(2):e0342380. doi: 10.1371/journal.pone.0342380 (PMC12875473; doi:10.1371/journal.pone.0342380)
Supplement: S6 Table — (DOCX) [file pone.0342380.s006.docx]

**Preliminary CFA Model Fit Indices**

S6_1_1. Preliminary CFA fit for Demands at Work (WLSMV)

| **Model** | **N (listwise)** | **χ²** | **df** | **p** | **χ²/df** | **CFI** | **TLI** | **NFI** | **RMSEA**  **(90% CI)** | **SRMR** |
| --- | --- | --- | --- | --- | --- | --- | --- | --- | --- | --- |
| 5-factor CFA | 225 | 220.53 | 80 | < .001 | 2.76 | 0.97 | 0.96 | 0.96 | 0.09 [0.08–0.10] | 0.08 |

S6_1_2. Reliability and convergent validity indices from the preliminary CFA for Demands at Work

| **Factor / subscale** | **Items (n)** | **Ordinal α** | **ω** | **AVE** |
| --- | --- | --- | --- | --- |
| **Quantitative Demands**  **(QD)** | 3 | 0.84 | 0.81 | 0.66 |
| **Work Pace**  **(WP)** | 3 | 0.87 | 0.85 | 0.73 |
| **Cognitive Demands**  **(CD)** | 3 | 0.74 | 0.7 | 0.53 |
| **Emotional & Decision Demands (EDD)** | 4 | 0.76 | 0.75 | 0.48 |
| **Demands for Hiding Emotions**  **(HE)** | 2 | 0.57 | 0.47 | 0.41 |

S6_1_3– Fornell–Larcker discriminant validity matrix for Demands at Work

|  | **QD** | **WP** | **CD** | **ED** | **HE** |
| --- | --- | --- | --- | --- | --- |
| **QD** | **0.81** |  |  |  |  |
| **WP** | 0.59 | **0.85** |  |  |  |
| **CD** | 0.38 | 0.45 | **0.72** |  |  |
| **ED** | 0.52 | 0.29 | 0.67 | **0.69** |  |
| **HE** | −0.11 | 0.2 | 0.38 | 0.28 | **0.64** |

S6_1_4. Heterotrait–Monotrait (HTMT) ratios of correlations for Demands at Work

| **Factor pair** | **HTMT** | **95% CI lower** | **95% CI upper** |
| --- | --- | --- | --- |
| QD – WP | 0.61 | 0.46 | 0.72 |
| QD – CD | 0.39 | 0.19 | 0.53 |
| QD – ED | 0.49 | 0.29 | 0.63 |
| QD – HE | 0.04 | 0.05 | 0.31 |
| WP – CD | 0.43 | 0.2 | 0.57 |
| WP – ED | 0.25 | 0.14 | 0.37 |
| WP – HE | 0.15 | 0.05 | 0.37 |
| CD – ED | 0.60 | 0.35 | 0.76 |
| CD – HE | 0.33 | 0.12 | 0.57 |
| ED – HE | 0.24 | 0.09 | 0.51 |

S6_2_1. Preliminary CFA fit for Work organization and Job Contents (WLSMV)

| **Model** | **N (listwise)** | **χ²** | **df** | **p** | **χ²/df** | **CFI** | **TLI** | **NFI** | **RMSEA (90% CI)** | **SRMR** |
| --- | --- | --- | --- | --- | --- | --- | --- | --- | --- | --- |
| 5-factor CFA | 221 | 68.98 | 55.00 | 0.10 | 1.25 | 1.00 | 1.00 | 0.99 | .034 (0.000–0.057) | 0.06 |

S6_2_2. Reliability and convergent validity indices from the preliminary CFA for Work organization and Job Contents

| **Factor / subscale** | **Items (n)** | **Ordinal α** | **ω** | **AVE** |
| --- | --- | --- | --- | --- |
| **Possibilities for Development (PD)** | 3 | 0.89 | 0.86 | 0.74 |
| **Meaning of Work (MW)** | 2 | 0.94 | 0.87 | 0.88 |
| **Workplace Autonomy (WA)** | 4 | 0.71 | 0.65 | 0.39 |
| **Variation of Work (VA)** | 2 | 0.80 | 0.77 | 0.71 |
| **Influence at Work (IN)** | 2 | 0.72 | 0.68 | 0.57 |

S6_2_3. Fornell–Larcker discriminant validity matrix for Work organization and Job Contents

| **Factor** | **PD** | **MW** | **WA** | **VA** | **IN** |
| --- | --- | --- | --- | --- | --- |
| **PD** | **0.86** |  |  |  |  |
| **MW** | 0.57 | **0.94** |  |  |  |
| **WA** | 0.25 | -0.02 | **0.62** |  |  |
| **VA** | 0.59 | 0.36 | 0.28 | **0.84** |  |
| **IN** | 0.36 | 0.29 | 0.49 | 0.27 | **0.75** |

S6_2_4. Heterotrait–Monotrait (HTMT) ratios of correlations for Work organization and Job Contents

| **Factor pair** | **HTMT** | **95% CI lower** | **95% CI upper** |
| --- | --- | --- | --- |
| PD– MW | 0.57 | 0.41 | 0.68 |
| PD– WA | 0.20 | 0.10 | 0.38 |
| PD– VA | 0.60 | 0.45 | 0.73 |
| PD– IN | 0.36 | 0.16 | 0.51 |
| MW – WA | 0.07 | 0.04 | 0.22 |
| MW – VA | 0.37 | 0.20 | 0.52 |
| MW – IN | 0.28 | 0.08 | 0.47 |
| WA – VA | 0.23 | 0.10 | 0.38 |
| WA – IN | 0.34 | 0.23 | 0.62 |
| VA – IN | 0.24 | 0.05 | 0.44 |

S6_3_1. Preliminary CFA fit for Interpersonal Relations and Leadership (WLSMV)

| **Model** | **N (listwise)** | **χ² (df)** | **p** | **χ²/df** | **CFI** | **TLI** | **NFI** | **RMSEA (90% CI)** | **SRMR** |
| --- | --- | --- | --- | --- | --- | --- | --- | --- | --- |
| 6-factor CFA | 220 | 207.26 (155) | 0.00 | 1.34 | 1.00 | 1.00 | 0.99 | 0.039 [0.024, 0.053] | 0.06 |

S6_3_2. Reliability and convergent validity indices from the CFA for Relations and Leadership

| **Factor / subscale** | **Items (n)** | **Ordinal α** | **ω** | **AVE** |
| --- | --- | --- | --- | --- |
| **Quality of Leadership**  **(QL)** | 5 | 0.89 | 0.88 | 0.65 |
| **Recognition**  **(RE)** | 3 | 0.97 | 0.93 | 0.92 |
| **Sense of Community at Work**  **(SW)** | 3 | 0.94 | 0.88 | 0.83 |
| **Role Clarity**  **(CL)** | 3 | 0.90 | 0.834 | 0.76 |
| **Role & Task Conflict**  **(RT)** | 3 | 0.85 | 0.80 | 0.65 |
| **Social Support from Colleagues**  **(SC)** | 3 | 0.79 | 0.77 | 0.64 |

S6_3_3. Fornell–Larcker discriminant validity matrix for Relations and Leadership

| **Factor** | **QL** | **RE** | **SW** | **CL** | **CO** | **SC** |
| --- | --- | --- | --- | --- | --- | --- |
| **QL** | **0.8** |  |  |  |  |  |
| **RE** | 0.74 | **0.96** |  |  |  |  |
| **SW** | 0.45 | 0.51 | **0.91** |  |  |  |
| **CL** | 0.58 | 0.67 | 0.5 | **0.87** |  |  |
| **CO** | −0.32 | −0.47 | −0.33 | −0.41 | **0.8** |  |
| **SC** | 0.5 | 0.58 | 0.7 | 0.52 | −0.38 | **0.8** |

S6_3_4. Heterotrait–Monotrait (HTMT) ratios of correlations for Relations and Leadership

| **Factor pair** | **HTMT** | **95% CI lower** | **95% CI upper** |
| --- | --- | --- | --- |
| QL – RE | 0.76 | 0.65 | 0.84 |
| QL – SW | 0.42 | 0.23 | 0.55 |
| QL – CL | 0.55 | 0.35 | 0.68 |
| QL – CO | 0.28 | 0.15 | 0.39 |
| QL – SC | 0.53 | 0.34 | 0.66 |
| RE – SW | 0.51 | 0.38 | 0.62 |
| RE – CL | 0.67 | 0.55 | 0.76 |
| RE – CO | 0.46 | 0.29 | 0.58 |
| RE – SC | 0.62 | 0.48 | 0.74 |
| SW – CL | 0.5 | 0.35 | 0.62 |
| SW – CO | 0.3 | 0.14 | 0.45 |
| SW – SC | 0.71 | 0.57 | 0.82 |
| CL – CO | 0.39 | 0.2 | 0.52 |
| CL – SC | 0.52 | 0.31 | 0.66 |
| CO – SC | 0.27 | 0.19 | 0.46 |

S6_4_1. Preliminary CFA fit for Work-Individual Interface (WLSMV)

| **Model** | **N (listwise)** | **χ²** | **df** | **p** | **χ²/df** | **CFI** | **TLI** | **NFI** | **RMSEA**  **(90% CI)** | **SRMR** |
| --- | --- | --- | --- | --- | --- | --- | --- | --- | --- | --- |
| 6-factor CFA | 225 | 309.67 | 194 | < .001 | 1.6 | 0.99 | 0.99 | 0.99 | 0.05 (0.04–0.06) | 0.07 |

S6_4_2. Reliability and convergent validity indices from the CFA for Work-Individual Interface

| **Factor / subscale** | **Items (n)** | **Ordinal α** | **ω** | **AVE** |
| --- | --- | --- | --- | --- |
| **Commitment to the Workplace**  **(CW)** | 5 | 0.9 | 0.88 | 0.68 |
| **Insecurity Over Working Conditions**  **(IW)** | 4 | 0.88 | 0.84 | 0.65 |
| **Quality of Work**  **(QW)** | 2 | 0.93 | 0.91 | 0.88 |
| **Insecurity Over Employment (JI)** | 3 | 0.82 | 0.77 | 0.62 |
| **Work-Life Conflict**  **(WF)** | 5 | 0.86 | 0.84 | 0.63 |
| **Work Engagement**  **(WE)** | 3 | 0.94 | 0.92 | 0.84 |

S6_4_3. Fornell–Larcker discriminant validity matrix for Work-Individual Interface

| **Factor** | **CW** | **IW** | **QW** | **JI** | **WLC** | **WE** |
| --- | --- | --- | --- | --- | --- | --- |
| **CW** | **0.82** |  |  |  |  |  |
| **IW** | 0.18 | **0.81** |  |  |  |  |
| **QW** | 0.26 | 0.11 | **0.94** |  |  |  |
| **JI** | 0.07 | 0.65 | 0.08 | **0.79** |  |  |
| **WLC** | 0.31 | 0.39 | 0.11 | 0.26 | **0.8** |  |
| **WE** | 0.68 | 0.19 | 0.32 | 0.18 | 0.43 | **0.92** |

S6_4_4. Heterotrait–Monotrait (HTMT) ratios of correlations for Work-Individual Interface

| **Factor pair** | **HTMT** | **95% CI lower** | **95% CI upper** |
| --- | --- | --- | --- |
| CW – IW | 0.21 | 0.09 | 0.32 |
| CW – QW | 0.24 | 0.07 | 0.42 |
| CW – JI | 0.13 | 0.05 | 0.25 |
| CW – WLC | 0.42 | 0.22 | 0.52 |
| CW – WE | 0.70 | 0.57 | 0.77 |
| IW – QW | 0.12 | 0.03 | 0.28 |
| IW – JI | 0.67 | 0.49 | 0.77 |
| IW – WLC | 0.38 | 0.18 | 0.53 |
| IW – WE | 0.25 | 0.09 | 0.39 |
| QW – JI | 0.00 | 0.03 | 0.18 |
| QW – WLC | 0.16 | 0.08 | 0.26 |
| QW – WE | 0.32 | 0.15 | 0.45 |
| JI – WLC | 0.30 | 0.13 | 0.45 |
| JI – WE | 0.24 | 0.06 | 0.36 |
| WLC – WE | 0.48 | 0.23 | 0.57 |

S6_5_1. Preliminary CFA fit for Social Capital (WLSMV)

| **Model** | **N (listwise)** | **χ² (df)** | **p** | **χ²/df** | **CFI** | **TLI** | **NFI** | **RMSEA (90% CI)** | **SRMR** |
| --- | --- | --- | --- | --- | --- | --- | --- | --- | --- |
| 3-factor CFA | 236 | 56.54 (32) | 0.005 | 1.77 | 0.997 | 0.996 | 0.99 | 0.057 [0.031, 0.081] | 0.052 |

S6_5_2. Reliability and convergent validity indices from the preliminary CFA for Social Capital

| **Factor / subscale** | **Items (n)** | **Ordinal α** | **ω** | **AVE** |
| --- | --- | --- | --- | --- |
| **Organizational Justice**  **(JU)** | 5 | 0.90 | 0.87 | 0.66 |
| **Horizontal Trust**  **(TE)** | 3 | 0.84 | 0.84 | 0.71 |
| **Vertical Trust**  **(TM)** | 2 | 0.82 | 0.79 | 0.74 |

S6_5_3. Fornell–Larcker discriminant validity matrix for Social Capital

| **Factor** | **JU** | **TE** | **TM** |
| --- | --- | --- | --- |
| **JU** | **0.81** |  |  |
| **TE** | 0.56 | **0.84** |  |
| **TM** | 0.77 | 0.55 | **0.86** |

S6_5_4. Heterotrait–Monotrait (HTMT) ratios of correlations for Social Capital

| **Factor pair** | **HTMT** | **95% CI lower** | **95% CI upper** |
| --- | --- | --- | --- |
| JU – TE | 0.59 | 0.43 | 0.72 |
| JU – TM | 0.77 | 0.67 | 0.85 |
| TE – TM | 0.59 | 0.43 | 0.73 |

S6_6_1. Preliminary CFA fit for Conflicts and Offensive Behaviors (WLSMV)

| **Model** | **N (listwise)** | **χ² (df)** | **p** | **χ²/df** | **CFI** | **TLI** | **NFI** | **RMSEA (90% CI)** | **SRMR** |
| --- | --- | --- | --- | --- | --- | --- | --- | --- | --- |
| 2-factor CFA | 242 | 5.98 (8) | 0.649 | 0.75 | 1.00 | 1.004 | 0.994 | 0.000 [0.000, 0.062] | 0.07 |

S6_6_2. Reliability and convergent validity indices from the preliminary CFA for Conflicts and Offensive Behaviors

| **Factor / subscale** | **Items (n)*** | **Ordinal α** | **ω** | **AVE** |
| --- | --- | --- | --- | --- |
| [**Workplace Behavioral Transgression**](https://link.springer.com/article/10.1007/s10869-019-09622-1)  **(WBT)** | 3 | 0.92 | 0.8 | 0.79 |
| **Violence and Harassment**  **(VH)** | 3 | 0.86 | 0.77 | 0.69 |

S6_6_3. Fornell–Larcker discriminant validity matrix for Conflicts and Offensive Behaviors

| **Factor** | **WBT** | **VH** |
| --- | --- | --- |
| WBT | **0.89** |  |
| VH | 0.14 | **0.83** |

S6_6_4. Heterotrait–Monotrait (HTMT) ratios of correlations for Conflicts and Offensive Behaviors

| **Factor pair** | **HTMT** | **95% CI lower** | **95% CI upper** |
| --- | --- | --- | --- |
| WBT – VH | 0.14 | 0.07 | 0.46 |

S6_7_1. Preliminary CFA fit for Health and Well-being (WLSMV)

| **Model** | **N (listwise)** | **χ² (df)** | **p** | **χ²/df** | **CFI** | **TLI** | **NFI** | **RMSEA (90% CI)** | **SRMR** |
| --- | --- | --- | --- | --- | --- | --- | --- | --- | --- |
| 5-factor CFA | 231 | 164.50 (109) | < .001 | 1.51 | 0.998 | 0.997 | 0.994 | 0.047 [0.032, 0.061] | 0.058 |

S6_7_2. Reliability and convergent validity indices from the preliminary CFA for Health and Well-being

| **Factor / subscale** | **Items (n)*** | **Ordinal α** | **ω** | **AVE** |
| --- | --- | --- | --- | --- |
| **Burnout**  **(BO)** | 3 | 0.95 | 0.92 | 0.87 |
| **Cognitive Well-being Assessment**  **(CWA)** | 5 | 0.9 | 0.87 | 0.67 |
| **Sleeping Troubles (SL)** | 4 | 0.91 | 0.91 | 0.77 |
| **Somatic Stress**  **(SO)** | 3 | 0.79 | 0.71 | 0.56 |
| **Stress**  **(ST)** | 2 | 0.92 | 0.87 | 0.85 |

S6_7_3. Fornell–Larcker discriminant validity matrix for Health and Well-being

| **Factor** | **BO** | **CWA** | **SL** | **SO** | **ST** |
| --- | --- | --- | --- | --- | --- |
| **BO** | **0.93** |  |  |  |  |
| **CWA** | 0.6 | **0.82** |  |  |  |
| **SL** | 0.6 | 0.55 | **0.88** |  |  |
| **SO** | 0.71 | 0.63 | 0.6 | **0.75** |  |
| **ST** | 0.72 | 0.67 | 0.58 | 0.77 | **0.92** |

S6_7_4. Heterotrait–Monotrait (HTMT) ratios of correlations for Health and Well-being

| **Factor pair** | **HTMT** | **95% CI lower** | **95% CI upper** |
| --- | --- | --- | --- |
| BO – CWA | 0.58 | 0.47 | 0.69 |
| BO – SL | 0.62 | 0.51 | 0.71 |
| BO – SO | 0.68 | 0.56 | 0.78 |
| BO – ST | 0.72 | 0.62 | 0.81 |
| CWA – SL | 0.57 | 0.43 | 0.67 |
| CWA – SO | 0.61 | 0.42 | 0.74 |
| CWA – ST | 0.67 | 0.56 | 0.76 |
| SL – SO | 0.58 | 0.43 | 0.71 |
| SL – ST | 0.61 | 0.49 | 0.70 |
| SO – ST | 0.76 | 0.64 | 0.84 |

S6_8_1. Preliminary CFA fit for Personality (WLSMV)

| **Model** | **N (listwise)** | **χ² (df)** | **p** | **χ²/df** | **CFI** | **TLI** | **NFI** | **RMSEA**  **(90% CI)** | **SRMR** |
| --- | --- | --- | --- | --- | --- | --- | --- | --- | --- |
| 2-factor CFA | 239 | 9.01 (8) | 0.342 | 1.13 | 0.999 | 0.999 | 0.994 | 0.023 [0.000, 0.082] | 0.04 |

S6_8_2. Reliability and convergent validity indices from the preliminary CFA for Personality

| **Factor / subscale** | **Items (n)*** | **Ordinal α** | **ω** | **AVE** |
| --- | --- | --- | --- | --- |
| Problem-Solving Self-Efficacy (PS) | 2 | 0.85 | 0.77 | 0.6 |
| Goal-Directed Self-Efficacy (GD) | 2 | 0.63 | 0.54 | 0.46 |

S6_8_3. Fornell–Larcker discriminant validity matrix for Personality

| **Factor** | **F_SE1** | **F_SE2** |
| --- | --- | --- |
| PS | **0.77** |  |
| GD | 0.74 | **0.68** |

S6_8_4. Heterotrait–Monotrait (HTMT) ratios of correlations for Personality

| **Factor pair** | **HTMT** | **95% CI lower** | **95% CI upper** |
| --- | --- | --- | --- |
| PS – GD | 0.75 | 0.57 | 0.94 |
