# Supplementary material for: Streamlining psychosocial risk assessment: An exploratory adaptation of the COPSOQ III for Flemish healthcare workers
Source: PLoS One. 2026 Feb 5;21(2):e0342380. doi: 10.1371/journal.pone.0342380 (PMC12875473; doi:10.1371/journal.pone.0342380)
Supplement: S7 Table — (DOCX) [file pone.0342380.s007.docx]

S7 Table. Mapping of COPSOQ III Dimensions to the Extended Flemish Version (Retained, Shortened, Merged, Split, Dropped)

| **Domain 1 — Demands at Work** | | | | |
| --- | --- | --- | --- | --- |
| **Original dimension (code)** | **Original items** | **EFA decision** | **Revised dimension (code)** | **Revised items** |
| Cognitive Demands (CD) | CD1, CD2, CD3, CD4 | **Shortened + 1 item reallocated** | Cognitive Demands (CD) | CD1, CD2, CD3 |
| Emotional Demands (ED) | ED1, ED2, ED3 | **Merged (with CD4) + renamed** | Emotional & Decision Demands (EDD) | ED1, ED2, ED3, **CD4** |
| Quantitative Demands (QD) | QD1, QD2, QD3, QD4 | **Shortened** (QD3 dropped) | Quantitative Demands (QD) | QD1, QD2, QD4 |
| Demands for Hiding Emotions (HE) | HE1, HE2, HE3, HE4 | **Shortened** (HE2, HE4 dropped) | Demands for Hiding Emotions (HE) | HE1, HE3 |
| Work Pace (WP) | WP1, WP2, WP3 | **Retained** | Work Pace (WP) | WP1, WP2, WP3 |
| Note:   - **Dropped items in this domain:** *QD3, HE2, HE4* - **ED + CD4 → Emotional & Decision Demands (EDD):** *CD4 (“difficult decisions”) clustered with the emotional demand items and showed a salient loading on the same factor (Table 1), indicating that decisional strain in this sample behaves as part of an emotional/decision-demand construct. Conceptually, difficult decisions in healthcare are often embedded in emotionally demanding situations, so the merged scale improves coherence while preserving key content.* - **QD3 dropped (QD shortened):** *In preliminary EFAs, QD3 showed weak and/or non-specific (cross-)loadings relative to the other quantitative demand items; removing it improved simple structure while retaining coverage of workload accumulation and time-pressure via QD1, QD2, and QD4.* - **HE shortened to HE1+HE3 (HE2, HE4 dropped):** *HE2 and HE4 did not meet the loading/cross-loading criteria in preliminary EFAs. We retained HE1 and HE3 because they capture the core “display-rule” aspect of emotional concealment in patient-facing work; however, the resulting two-item scale is interpreted cautiously due to its brevity.* | | | | |
| **Domain 2 — Work Organization and Job Contents** | | | | |
| **Original dimension (code)** | **Original items** | **EFA decision** | **Revised dimension (code)** | **Revised items** |
| Possibilities for Development (PD) | PD1, PD2, PD3 | **Retained** | Possibilities for Development (PD) | PD1, PD2, PD3 |
| Influence at Work (IN) | IN1, IN2, IN3, IN4, IN5, IN6 | **Split + shortened** | Influence at Work (IN) | IN4, IN6 |
| Control Over Working Time (CT) | CT1, CT2, CT3, CT4, CT5 | **Dimension removed as stand-alone; 1 item reallocated** | Workplace Autonomy (WA) | IN2, IN3, IN5, CT4 |
| Influence at Work (IN) | IN1, IN2, IN3, IN4, IN5, IN6 | **Part reallocated** |  |  |
| Variation of Work (VA) | VA1, VA2 | **Retained** | Variation of Work (VA) | VA1, VA2 |
| Meaning of Work (MW) | MW1, MW2 | **Retained** | Meaning of Work (MW) | MW1, MW2 |
| **Note:**   - **Dropped items in this domain:** IN1, CT1, CT2, CT3, CT5 - **CT dimension removed; CT4 + IN2/IN3/IN5 → Workplace Autonomy (WA):** *CT items did not form a stable, coherent factor in preliminary EFAs, but CT4 loaded together with IN2/IN3/IN5 (Table 1), forming a consistent “day-to-day autonomy” factor. The resulting WA construct aligns theoretically with operational autonomy (control over small scheduling/coordination decisions) that is salient in healthcare work.* - **Influence at Work shortened (IN4+IN6 retained; IN1 dropped):** *IN4 and IN6 defined a clear “influence over tasks/methods” factor (Table 1), whereas IN1 showed weak or non-specific behavior in preliminary EFAs. This yields a more focused influence construct with minimal cross-loading.* | | | | |
| **Domain 3 — Interpersonal Relations and Leadership** | | | | |
| **Original dimension (code)** | **Original items** | **EFA decision** | **Revised dimension (code)** | **Revised items** |
| Predictability (PR) | PR1, PR2 | **Dropped** | — | — |
| Quality of Leadership (QL) | QL1, QL2, QL3, QL4 | **Expanded (merge)** | Quality of Leadership (QL) | QL1–QL4 + **SS3** |
| Social Support from Supervisor (SS) | SS1, SS2, SS3 | **Split: 1 item merged, 2 dropped** |  |  |
| Recognition (RE) | RE1, RE2, RE3 | **Retained** | Recognition (RE) | RE1, RE2, RE3 |
| Role Clarity (CL) | CL1, CL2, CL3 | **Retained** | Role Clarity (CL) | CL1, CL2, CL3 |
| Role Conflicts (CO) | CO1, CO2 | **Merged** | Role & Task Conflict (RT) | CO1, CO2, **IT1** |
| Illegitimate Tasks (IT) | IT1 | **Merged** |  |  |
| Sense of Community at Work (SW) | SW1, SW2, SW3 | **Retained** | Sense of Community at Work (SW) | SW1, SW2, SW3 |
| Social Support from Colleagues (SC) | SC1, SC2, SC3 | **Retained** | Social Support from Colleagues (SC) | SC1, SC2, SC3 |
| Note:   - **Dropped items in this domain:** PR1, PR2, SS1, SS2 - **SS3 merged with QL → Quality of Leadership (QL); SS1–SS2 dropped:** *SS3 (“superior talks with you about how well you carry out your work”) loaded with the leadership items (Table 1), indicating it functions as performance feedback/leadership behavior rather than generic support. SS1–SS2 did not meet loading/cross-loading criteria in preliminary EFAs, so the revised QL scale captures supportive, feedback-oriented leadership more coherently.* - **CO1+CO2+IT1 → Role & Task Conflict (RT):** *CO and IT items formed a single factor with salient loadings (Table 1), suggesting that contradictory demands and “unnecessary” tasks co-occur as a unified strain dimension in this sample. Conceptually, both reflect impaired role/task legitimacy and competing expectations.* - **Predictability (PR1–PR2) dropped:** *Predictability items did not emerge as a distinct, stable factor in preliminary EFAs (weak or cross-loadings), and therefore were removed to preserve a cleaner factor structure.* | | | | |
| **Domain 4 — Work–Individual Interface** | | | | |
| **Original dimension (code)** | **Original items** | **EFA decision** | **Revised dimension (code)** | **Revised items** |
| Commitment to the Workplace (CW) | CW1–CW5 | **Retained** | Commitment to the Workplace (CW) | CW1–CW5 |
| Work Engagement (WE) | WE1–WE3 | **Retained** | Work Engagement (WE) | WE1–WE3 |
| Insecurity Over Employment (JI) | JI1–JI3 | **Retained** | Insecurity Over Employment (JI) | JI1–JI3 |
| Insecurity Over Working Conditions (IW) | IW1–IW5 | **Shortened** (IW5 dropped) | Insecurity Over Working Conditions (IW) | IW1–IW4 |
| Quality of Work (QW) | QW1, QW2 | **Retained** | Quality of Work (QW) | QW1, QW2 |
| Work Life Conflict (WF) | WF1–WF5 | **Retained** | Work Life Conflict (WF) | WF1–WF5 |
| Job Satisfaction (JS) | JS1–JS5 | **Dropped** | — | — |
| **Note:**   - **Dropped items in this domain:** IW5, JS1–JS5 - **Job Satisfaction (JS1–JS5) dropped:** *In the preliminary EFAs, job satisfaction items displayed substantial cross-loadings and weak factor loadings, preventing a clean and interpretable standalone factor. Consistent with the exploratory aim, we prioritized constructs that demonstrated clearer, more stable measurement properties in this sample.* - **IW5 dropped (IW shortened to IW1–IW4):** *IW5 (“good prospects”) behaved differently from the threat-focused insecurity items (IW1–IW4) in preliminary EFAs (non-salient or cross-loading), so it was removed to maintain conceptual and statistical consistency of the insecurity construct.* | | | | |
| **Domain 5 — Social Capital** | | | | |
| **Original dimension (code)** | **Original items** | **EFA decision** | **Revised dimension (code)** | **Revised items** |
| Organizational Justice (JU) | JU1–JU4 | **Retained + expanded** | Organizational Justice (JU) | JU1–JU4 + **TM4** |
| Vertical Trust (TM) | TM1, TM2, TM3, TM4 | **Reallocated** |  |  |
| Vertical Trust (TM) | TM1, TM2, TM3, TM4 | **Split** | Vertical Trust (TM) | TM1, TM2 |
|  |  | **Reallocated** | Horizontal Trust (TE) | TE1, TE2, **TM3** |
| Horizontal Trust (TE) | TE1, TE2, TE3 | **Shortened + merged** |  |  |
| Note:   - **Dropped items in this domain:** TE3 - **TM4 merged with JU → Organizational Justice (JU):** *TM4 (voice/expression) loaded with the justice items (Table 1), consistent with procedural justice and employee voice as part of fair decision-making. This merger improves coherence of the justice construct in this organizational context.* - **TE1+TE2+TM3 → Horizontal Trust (TE); TE3 dropped:** *TE1/TE2 and TM3 all tap “information withholding/sharing” and loaded together (Table 1). TE3 (“employees trust each other in general”) showed a weak primary factor loading in the preliminary EFAs and was therefore excluded, yielding a more content-homogeneous trust factor.* - **Vertical Trust (TM) shortened to TM1–TM2:** *TM1–TM2 provided a clear management-trust factor with salient loadings (Table 1), while TM3 and TM4 aligned better with information-withholding and justice/voice, respectively, and were reallocated accordingly.* | | | | |
| **Domain 6 — Conflicts and Offensive Behaviors** | | | | |
| **Original dimension (code)** | **Original items** | **EFA decision** | **Revised dimension (code)** | **Revised items** |
| Bullying (BU) | BU1, BU2 | **Merged** | Workplace Behavioral Transgression (WBT) | BU1, BU2, **UT1** |
| Unpleasant Teasing (UT) | UT1 | **Merged** |  |  |
| Sexual Harassment (SH) | SH1 | **Merged** | Violence and Harassment (VH) | SH1, TV1, PV1 |
| Threats of Violence (TV) | TV1 | **Merged** |  |  |
| Physical Violence (PV) | PV1 | **Merged** |  |  |
| Gossip and Slander (GS) | GS1 | **Dropped** | — | — |
| Conflicts and Quarrels (CQ) | CQ1 | **Dropped** | — | — |
| Harassment in Social Media (HSM) | HSM1 | **Dropped** | — | — |
| **Note:**   - **Dropped items in this domain:** GS1, CQ1, HSM1 - **BU + UT → Workplace Behavioral Transgression (WBT):** *Bullying and unpleasant teasing items loaded on a single factor (Table 1), indicating a unified “behavioral transgression” construct reflecting hostile interpersonal behaviors.* - **PV + TV + SH → Violence and Harassment (VH):** *Physical violence, threats, and sexual harassment items loaded together (Table 1), supporting a coherent violence/harassment dimension.* - **GS1, CQ1, HSM1 dropped:** *These single-item indicators were excluded because they showed unstable measurement behavior—most notably weak factor loadings in preliminary EFAs—so as to avoid unreliable single-item factors.* | | | | |
| **Domain 7 — Health and Well-Being** | | | | |
| **Original dimension (code)** | **Original items** | **EFA decision** | **Revised dimension (code)** | **Revised items** |
| Burnout (BO) | BO1–BO4 | **Shortened** (BO3 dropped) | Burnout (BO) | BO1, BO2, BO4 |
| Stress (ST) | ST1–ST3 | **Shortened** (ST1 dropped) | Stress (ST) | ST2, ST3 |
| Sleeping Troubles (SL) | SL1–SL4 | **Retained** | Sleeping Troubles (SL) | SL1–SL4 |
| Somatic Stress (SO) | SO1–SO4 | **Shortened** (SO4 dropped) | Somatic Stress (SO) | SO1–SO3 |
| Cognitive Stress (CS) | CS1–CS4 | **Merged + renamed** | Cognitive Well-being Assessment (CWA) | CS1–CS4 + **DS3** |
| Depressive Symptoms (DS) | DS1–DS4 | **Dropped as stand-alone; 1 item reallocated** |  |  |
| Self-Related Health (GH) | GH1 | **Dropped** | — | — |
| Note:   - **Dropped items in this domain:** BO3, ST1, SO4, DS1, DS2, DS4, GH1 - **CS1–CS4 + DS3 → Cognitive Well-being Assessment (CWA); DS1/DS2/DS4 dropped:** *DS3 clustered with the cognitive stress items and showed a salient loading on the same factor (Table 1), suggesting that guilt/burdensome thoughts operate as part of cognitive strain in this sample. Other depressive items did not meet loading/cross-loading criteria in preliminary EFAs and were removed to preserve a coherent cognitive-strain construct.* - **BO shortened (BO3 dropped), ST shortened (ST1 dropped), SO shortened (SO4 dropped):** *The excluded items showed weak or non-specific behavior in preliminary EFAs; retained items formed cleaner factors with salient loadings (Table 1).* - **GH1 dropped:** *Self-rated health was removed from the EFA-derived measurement model because it showed a weak factor loading in the preliminary EFAs.* | | | | |
| **Domain 8 — Personality** | | | | |
| **Original dimension (code)** | **Original items** | **EFA decision** | **Revised dimension (code)** | **Revised items** |
| Self-Efficacy (SE) | SE1–SE6 | **Split** | Problem-Solving Self-Efficacy (PS) | SE1, SE4, SE5, SE6 |
| Self-Efficacy (SE) | SE1–SE6 | **Split** | Goal-Directed Self-Efficacy (GD) | SE2, SE3 |
| Note:   - **Self-Efficacy split → PS vs GD:** *Parallel analysis and EFA supported two related but distinguishable self-efficacy facets (Table 1): problem-solving confidence (PS) versus goal/persistence-oriented confidence (GD). This split improves conceptual specificity while maintaining coverage of the original construct.* | | | | |
