# Supplementary material for: Streamlining psychosocial risk assessment: An exploratory adaptation of the COPSOQ III for Flemish healthcare workers
Source: PLoS One. 2026 Feb 5;21(2):e0342380. doi: 10.1371/journal.pone.0342380 (PMC12875473; doi:10.1371/journal.pone.0342380)
Supplement: S1 File — (PDF) [file pone.0342380.s008.pdf]

S1 Questionnaire. Extended Flemish version of COPSOQ III (adapted for healthcare workers)

This questionnaire is an adapted Flemish version of the Copenhagen Psychosocial Questionnaire III (COPSOQ III), originally developed by the COPSOQ International Network (Clays et al., Ghent University, Securex). The adaptation was performed to evaluate psychosocial risks in healthcare workers in Flanders. The version presented here includes the final factor structure (30 dimensions) validated in this study.

This instrument is made available under the Creative Commons Attribution 4.0 International License (CC BY 4.0).

Citation of original source:

Clays E, Vyvey K, Bolliger L, De Meester M, Kiss P. Reliability and validity of the COPSOQ III (Copenhagen Psychosocial Questionnaire) in Flemish healthcare workers. Ghent University & Securex, Belgium. (https://www.copsoq-network.org/validation-studies, chrome-extension://efaidnbmnnnibpcajpcglclefindmkaj/https://www.copsoq-network.org/assets/Uploads/VYVEY-2017.pdf)

Demands at Work	Questions	Answers
	Cognitive Demands (CD)	
CD1	Do you have to keep your eyes on lots of things while you work?	always (4), often (3), sometimes (2), seldom (1), never/hardly ever (0)
CD2	Does your work require that you remember at lot of things?	always (4), often (3), sometimes (2), seldom (1), never/hardly ever (0)
CD3	Does your work demand that you are good at coming up with new ideas?	always (4), often (3), sometimes (2), seldom (1), never/hardly ever (0)
CD4	Does your work require you to make difficult decisions?	always (4), often (3), sometimes (2), seldom (1), never/hardly ever (0)
	Quantitative Demands (QD)	
QD1	Is your workload unevenly distributed so it piles up?	always (4), often (3), sometimes (2), seldom (1), never/hardly ever (0)
QD2	How often do you not have time to complete all your work tasks?	always (4), often (3), sometimes (2), seldom (1), never/hardly ever (0)
QD3	Do you get behind with your work?	always (0), often (1), sometimes (2), seldom (3), never/hardly ever (4)
QD4	Do you have enough time for your work tasks?	always (4), often (3), sometimes (2), seldom

	(reversed scoring)	(1), never/hardly ever (0)
Emotional Demands (ED)		
ED1	Does your work put you in emotionally disturbing situations?	always (4), often (3), sometimes (2), seldom (1), never/hardly ever (0)
ED2	Do you have to deal with other people's personal problems as part of your work?	to a very large extent (4), to a large extent (3), somewhat (2), to a small extent (1), to a very small extent (0)
ED3	Is your work emotionally demanding?	always (4), often (3), sometimes (2), seldom (1), never/hardly ever (0)
HE2	Does your work require that you hide your feelings?	to a very large extent (4), to a large extent (3), somewhat (2), to a small extent (1), to a very small extent (0)
HE4	Does your work require that you do not state your opinion?	always (4), often (3), sometimes (2), seldom (1), never/hardly ever (0)
Ethical Conduct Expectations (ECE)		
HE1	Are you required to treat everyone equally, even if you do not feel like it?	always (4), often (3), sometimes (2), seldom (1), never/hardly ever (0)
HE3	Are you required to be kind and open towards everyone – regardless of how they behave towards you?	to a very large extent (4), to a large extent (3), somewhat (2), to a small extent (1), to a very small extent (0)
Work Pace (WP)		
WP1	Do you have to work very fast?	always (4), often (3), sometimes (2), seldom (1), never/hardly ever (0)
WP2	Do you work at a high pace throughout the day?	to a very large extent (4), to a large extent (3), somewhat (2), to a small extent (1), to a very small extent (0)
WP3	Is it necessary to keep working at a high pace?	to a very large extent (4), to a large extent (3), somewhat (2), to a small extent (1), to a very small extent (0)
Work Organization and Job Contents	Questions	Answers
Development and Meaning (DM)		
MW1	Is your work meaningful?	to a very large extent (4), to a large extent (3), somewhat (2), to a small extent (1), to a very small extent (0)
MW2	Do you feel that the work you do is important?	to a very large extent (4), to a large extent (3), somewhat (2), to a small extent (1), to a very small extent (0)
PD1	Do you have the possibility of learning new things through your work?	to a very large extent (4), to a large extent (3), somewhat (2), to a small extent (1), to a very small extent (0)
PD2	Can you use your skills or expertise in your work?	to a very large extent (4), to a large extent (3), somewhat (2), to a small extent (1), to a very small extent (0)
PD3	Does your work give you the opportunity to develop your skills?	to a very large extent (4), to a large extent (3), somewhat (2), to a small extent (1), to a very small extent (0)
Workplace Autonomy (WA)		
IN2	Do you have a say in choosing who you work with?	always (4), often (3), sometimes (2), seldom (1), never/hardly ever (0)
IN3	Can you influence the amount of work assigned to you?	always (4), often (3), sometimes (2), seldom (1), never/hardly ever (0)
CT4	If you have some private business is it possible for	always (4), often (3), sometimes (2), seldom

	you to leave your piece of work for half an hour without special permission?	(1), never/hardly ever (0)
CT5	Do you have to do overtime? *(reversed scoring)*	*always (0), often (1), sometimes (2), seldom (3), never/hardly ever (4)*
Influence at Work (IN)		
IN4	Do you have any influence on what you do at work?	always (4), often (3), sometimes (2), seldom (1), never/hardly ever (0)
IN5	Can you influence how quickly you work?	always (4), often (3), sometimes (2), seldom (1), never/hardly ever (0)
IN6	Do you have any influence on HOW you do your work?	always (4), often (3), sometimes (2), seldom (1), never/hardly ever (0)
Control over Working Time (CT)		
CT1	Can you decide when to take a break?	always (4), often (3), sometimes (2), seldom (1), never/hardly ever (0)
CT3	Can you leave your work to have a chat with a colleague?	always (4), often (3), sometimes (2), seldom (1), never/hardly ever (0)
Variation of Work (VA)		
VA1	Is your work varied?	always (4), often (3), sometimes (2), seldom (1), never/hardly ever (0)
VA2	Do you have to do the same thing over and over again? (reversed scoring)	always (0), often (1), sometimes (2), seldom (3), never/hardly ever (4)
Interpersonal Relations and Leadership	Questions	Answers
Quality of Vertical Interpersonal Relations (QVIR)		
PR1	At your place of work, are you informed well in advance concerning for example important decisions, changes or plans for the future?	to a very large extent (4), to a large extent (3), somewhat (2), to a small extent (1), to a very small extent (0)
PR2	Do you receive all the information you need in order to do your work well?	to a very large extent (4), to a large extent (3), somewhat (2), to a small extent (1), to a very small extent (0)
RE1	Is your work recognized and appreciated by the management?	to a very large extent (4), to a large extent (3), somewhat (2), to a small extent (1), to a very small extent (0)
RE3	Are you treated fairly at your workplace?	to a very large extent (4), to a large extent (3), somewhat (2), to a small extent (1), to a very small extent (0)
QL1	makes sure that the members of staff has good development opportunities?	to a very large extent (4), to a large extent (3), somewhat (2), to a small extent (1), to a very small extent (0)
QL2	gives high priority to job satisfaction?	to a very large extent (4), to a large extent (3), somewhat (2), to a small extent (1), to a very small extent (0)
QL3	is good at work planning?	to a very large extent (4), to a large extent (3), somewhat (2), to a small extent (1), to a very small extent (0)
QL4	is good at solving conflicts?	to a very large extent (4), to a large extent (3), somewhat (2), to a small extent (1), to a very small extent (0)
SS1	How often is your immediate superior willing to listen to your problems at work, if needed?	always (4), often (3), sometimes (2), seldom (1), never/hardly ever (0)
SS2	How often do you get help and support from your immediate superior, if needed?	always (4), often (3), sometimes (2), seldom (1), never/hardly ever (0)
SS3	How often does your immediate superior talk with you about how well you carry out your work?	always (4), often (3), sometimes (2), seldom (1), never/hardly ever (0)
Sense of community at work (SW)		
SW1	Is there a good atmosphere between you and your colleagues?	always (4), often (3), sometimes (2), seldom (1), never/hardly ever (0)
SW2	Is there good co-operation between the colleagues at work?	always (4), often (3), sometimes (2), seldom (1), never/hardly ever (0)
SW3	Do you feel part of a community at your place of	always (4), often (3), sometimes (2), seldom

	work?	(1), never/hardly ever (0)
Role & Task Conflict (RT)		
CO1	Are contradictory demands placed on you at work?	to a very large extent (4), to a large extent (3), somewhat (2), to a small extent (1), to a very small extent (0)
CO2	Do you sometimes have to do things which ought to have been done in a different way?	to a very large extent (4), to a large extent (3), somewhat (2), to a small extent (1), to a very small extent (0)
IT1	Do you sometimes have to do things which seem to be unnecessary?	to a very large extent (4), to a large extent (3), somewhat (2), to a small extent (1), to a very small extent (0)
Role Clarity (CL)		
CL1	Does your work have clear objectives?	to a very large extent (4), to a large extent (3), somewhat (2), to a small extent (1), to a very small extent (0)
CL2	Do you know exactly which areas are your responsibility?	to a very large extent (4), to a large extent (3), somewhat (2), to a small extent (1), to a very small extent (0)
CL3	Do you know exactly what is expected of you at work?	to a very large extent (4), to a large extent (3), somewhat (2), to a small extent (1), to a very small extent (0)
Work-Individual Interface	Questions	Answers
Commitment to the Workplace (CW)		
CW1	Do you enjoy telling others about your place of work?	to a very large extent (4), to a large extent (3), somewhat (2), to a small extent (1), to a very small extent (0)
CW2	Do you feel that your place of work is of great importance to you?	to a very large extent (4), to a large extent (3), somewhat (2), to a small extent (1), to a very small extent (0)
CW3	Would you recommend other people to apply for a position at your workplace?	to a very large extent (4), to a large extent (3), somewhat (2), to a small extent (1), to a very small extent (0)
CW4	How often do you consider looking for work elsewhere? *(reversed scoring)*	*always (0), often (1), sometimes (2), seldom (3), never/hardly ever (4)*
CW5	Are you proud of being part of this organization?	to a very large extent (4), to a large extent (3), somewhat (2), to a small extent (1), to a very small extent (0)
Work Engagement (WE)		
WE1	At my work, I feel bursting with energy.	never (0), seldom (1), sometimes (2), often (3), always (4)
WE2	I am enthusiastic about my job.	never (0), seldom (1), sometimes (2), often (3), always (4)
WE3	I am immersed in my work.	never (0), seldom (1), sometimes (2), often (3), always (4)
Insecurity Over Employment (JI)		
JI1	Are you worried about becoming unemployed?	to a very large extent (4), to a large extent (3), somewhat (2), to a small extent (1), to a very small extent (0)
JI2	Are you worried about new technology making you redundant?	to a very large extent (4), to a large extent (3), somewhat (2), to a small extent (1), to a very small extent (0)
JI3	Are you worried about it being difficult for you to find another job if you became unemployed?	to a very large extent (4), to a large extent (3), somewhat (2), to a small extent (1), to a very small extent (0)
Quality of Work (QW)		
QW1	To what extent do you find it possible to perform your work tasks at a satisfactory quality?	to a very large extent (4), to a large extent (3), somewhat (2), to a small extent (1), to a very small extent (0)
QW2	Are you satisfied with the quality of the work performed at your workplace?	to a very large extent (4), to a large extent (3), somewhat (2), to a small extent (1), to a

		very small extent (0)
Job Satisfaction (JS)		
JS1	your work prospects?	very satisfied (4), satisfied (3), neither/nor (2), unsatisfied (1), very unsatisfied (0)
JS2	the physical working conditions?	very satisfied (4), satisfied (3), neither/nor (2), unsatisfied (1), very unsatisfied (0)
JS3	the way your abilities are used?	very satisfied (4), satisfied (3), neither/nor (2), unsatisfied (1), very unsatisfied (0)
JS4	your job as a whole, everything taken into consideration?	very satisfied (4), satisfied (3), neither/nor (2), unsatisfied (1), very unsatisfied (0)
Work-life Conflict (WF)		
WF1	Are there times when you need to be at work and at home at the same time?	always (4), often (3), sometimes (2), seldom (1), never/hardly ever (0)
The next four questions concern the ways in which your work affects your private life:		
WF2	Do you feel that your work drains so much of your energy that it has a negative effect on your private life?	to a very large extent (4), to a large extent (3), somewhat (2), to a small extent (1), to a very small extent (0)
WF3	Do you feel that your work takes so much of your time that it has a negative effect on your private life?	to a very large extent (4), to a large extent (3), somewhat (2), to a small extent (1), to a very small extent (0)
WF4	The demands of my work interfere with my private and family life	to a very large extent (4), to a large extent (3), somewhat (2), to a small extent (1), to a very small extent (0)
WF5	Due to work-related duties, I have to make changes to my plans for private and family activities.	to a very large extent (4), to a large extent (3), somewhat (2), to a small extent (1), to a very small extent (0)
Insecurity Over Working Conditions (IW)		
IW1	Are you worried about being transferred to another job against your will?	to a very large extent (4), to a large extent (3), somewhat (2), to a small extent (1), to a very small extent (0)
IW2	Are you worried about your working tasks being changed against your will?	to a very large extent (4), to a large extent (3), somewhat (2), to a small extent (1), to a very small extent (0)
IW3	Are you worried about the timetable being changed (shift, weekdays, time to enter and leave ...) against your will?	to a very large extent (4), to a large extent (3), somewhat (2), to a small extent (1), to a very small extent (0)
IW4	Are you worried about a decrease in your salary (reduction, variable pay being introduced ...)?	to a very large extent (4), to a large extent (3), somewhat (2), to a small extent (1), to a very small extent (0)
Social Capital	Questions	Answers
Vertical Trust (VT)		
TM1	Does the management trust the employees to do their work well?	to a very large extent (4), to a large extent (3), somewhat (2), to a small extent (1), to a very small extent (0)
TM2	Can the employees trust the information that comes from the management?	to a very large extent (4), to a large extent (3), somewhat (2), to a small extent (1), to a very small extent (0)
TM4	Are the employees able to express their views and feelings?	to a very large extent (4), to a large extent (3), somewhat (2), to a small extent (1), to a very small extent (0)
JU1	Are conflicts resolved in a fair way?	to a very large extent (4), to a large extent (3), somewhat (2), to a small extent (1), to a very small extent (0)
JU2	Are employees appreciated when they have done a good job?	to a very large extent (4), to a large extent (3), somewhat (2), to a small extent (1), to a very small extent (0)
JU3	Are all suggestions from employees treated seriously by the management?	to a very large extent (4), to a large extent (3), somewhat (2), to a small extent (1), to a very small extent (0)
JU4	Is the work distributed fairly?	to a very large extent (4), to a large extent (3), somewhat (2), to a small extent (1), to a

		very small extent (0)
Overall Trust in the Organization (OT)		
TE1	Do the employees withhold information from each other? *(reversed scoring)*	to a very large extent (0), to a large extent (1), somewhat (2), to a small extent (3), to a very small extent (4)
TE2	Do the employees withhold information from the management? *(reversed scoring)*	to a very large extent (0), to a large extent (1), somewhat (2), to a small extent (3), to a very small extent (4)
TE3	Do the employees in general trust each other?	to a very large extent (4), to a large extent (3), somewhat (2), to a small extent (1), to a very small extent (0)
TM3	Does the management withhold important information from the employees? (reversed scoring)	to a very large extent (0), to a large extent (1), somewhat (2), to a small extent (3), to a very small extent (4)
Conflicts and Offensive Behaviors	Questions	Answers
Workplace Behavioral Transgression (WBT)		
GS1	Have you been exposed to gossip and slander at your workplace during the last 12 months?	Yes, daily. Yes, weekly. Yes, monthly. Yes, a few times. Yes, once. No.
CQ1	Have you been involved in quarrels or conflicts at your workplace during the last 12 months?	Yes, daily. Yes, weekly. Yes, monthly. Yes, a few times. Yes, once. No.
UT1	Have you been exposed to unpleasant teasing at your workplace during the last 12 months?	Yes, daily. Yes, weekly. Yes, monthly. Yes, a few times. Yes, once. No.
BU1	Have you been exposed to bullying at your workplace during the last 12 months?	Yes, daily. Yes, weekly. Yes, monthly. Yes, a few times. Yes, once. No.
BU2	Have you been unjustly criticized, bullied or shown up in front of others during the last 12 months?	Yes, daily. Yes, weekly. Yes, monthly. Yes, a few times. Yes, once. No.
Violence and Harassment (VH)		
HSM1	Have you been exposed to work-related harassment on the social media (e.g. Facebook), by e-mail or text messages during the last 12 months?	Yes, daily. Yes, weekly. Yes, monthly. Yes, a few times. Yes, once. No.
SH1	Have you been exposed to undesired sexual attention at your workplace during the last 12 months?	Yes, daily. Yes, weekly. Yes, monthly. Yes, a few times. Yes, once. No.
TV1	Have you been exposed to threats of violence at your workplace during the last 12 months?	Yes, daily. Yes, weekly. Yes, monthly. Yes, a few times. Yes, once. No.
PV1	Have you been exposed to physical violence at your workplace during the last 12 months?	Yes, daily. Yes, weekly. Yes, monthly. Yes, a few times. Yes, once. No.
Health and Well-Being	Questions	Answers
Tension and Exhaustion (TE)		
Top page: These questions are about how you have been during the last 5 weeks.		
BO1	How often have you felt worn out?	all the time (4), a large part of the time (3), part of the time (2), a small part of the time (1), not at all (0)
BO2	How often have you been physically exhausted?	all the time (4), a large part of the time (3), part of the time (2), a small part of the time (1), not at all (0)
BO3	How often have you been emotionally exhausted?	all the time (4), a large part of the time (3), part of the time (2), a small part of the time (1), not at all (0)
BO4	How often have you felt tired?	all the time (4), a large part of the time (3), part of the time (2), a small part of the time (1), not at all (0)
ST1	How often have you had problems relaxing?	all the time (4), a large part of the time (3), part of the time (2), a small part of the time (1), not at all (0)

ST2	How often have you been irritable?	all the time (4), a large part of the time (3), part of the time (2), a small part of the time (1), not at all (0)
ST3	How often have you been tense?	all the time (4), a large part of the time (3), part of the time (2), a small part of the time (1), not at all (0)
SO4	How often have you had tension in various muscles?	all the time (4), a large part of the time (3), part of the time (2), a small part of the time (1), not at all (0)
DS1	How often have you felt sad?	all the time (4), a large part of the time (3), part of the time (2), a small part of the time (1), not at all (0)
Cognitive Well-being Assessment (CWA)		
Top page: These questions are about how you have been during the last 5 weeks.		
CS1	How often have you had problems concentrating?	all the time (4), a large part of the time (3), part of the time (2), a small part of the time (1), not at all (0)
CS2	How often have you found it difficult to think clearly?	all the time (4), a large part of the time (3), part of the time (2), a small part of the time (1), not at all (0)
CS3	How often have you had difficulty in taking decisions?	all the time (4), a large part of the time (3), part of the time (2), a small part of the time (1), not at all (0)
CS4	How often have you had difficulty with remembering?	all the time (4), a large part of the time (3), part of the time (2), a small part of the time (1), not at all (0)
DS2	How often have you lacked self-confidence?	all the time (4), a large part of the time (3), part of the time (2), a small part of the time (1), not at all (0)
DS3	How often have you had a bad conscience or felt guilty?	all the time (4), a large part of the time (3), part of the time (2), a small part of the time (1), not at all (0)
Sleeping Troubles (SL)		
Top page: These questions are about how you have been during the last 4 weeks.		
SL1	How often have you slept badly and restlessly?	all the time (4), a large part of the time (3), part of the time (2), a small part of the time (1), not at all (0)
SL2	How often have you found it hard to go to sleep?	all the time (4), a large part of the time (3), part of the time (2), a small part of the time (1), not at all (0)
SL3	How often have you woken up too early and not been able to get back to sleep?	all the time (4), a large part of the time (3), part of the time (2), a small part of the time (1), not at all (0)
SL4	How often have you woken up several times and found it difficult to get back to sleep?	all the time (4), a large part of the time (3), part of the time (2), a small part of the time (1), not at all (0)
Somatic Stress (SO)		
Top page: These questions are about how you have been during the last 5 weeks.		
SO1	How often have you had stomach ache?	all the time (4), a large part of the time (3), part of the time (2), a small part of the time (1), not at all (0)
SO2	How often have you had a headache?	all the time (4), a large part of the time (3), part of the time (2), a small part of the time (1), not at all (0)
SO3	How often have you had palpitations?	all the time (4), a large part of the time (3), part of the time (2), a small part of the time (1), not at all (0)
Personality	Questions	Answers
Self-efficacy (SE)		

Top of page: How well do these descriptions fit on you as a person?		
SE1	I am always able to solve difficult problems, if I try hard enough.	fits perfectly (3), fits quite well (2), fits a little bit (1), does not fit (0)
SE2	If people work against me, I find a way of achieving what I want.	fits perfectly (3), fits quite well (2), fits a little bit (1), does not fit (0)
SE3	It is easy for me to stick to my plans and reach my objectives.	fits perfectly (3), fits quite well (2), fits a little bit (1), does not fit (0)
SE4	I feel confident that I can handle unexpected events.	fits perfectly (3), fits quite well (2), fits a little bit (1), does not fit (0)
SE5	When I have a problem, I can usually find several ways of solving it.	fits perfectly (3), fits quite well (2), fits a little bit (1), does not fit (0)
SE6	Regardless of what happens, I usually manage.	fits perfectly (3), fits quite well (2), fits a little bit (1), does not fit (0)
